# Emerging Flexible Thermally Conductive Films: Mechanism, Fabrication, Application

**DOI:** 10.1007/s40820-022-00868-8

**Published:** 2022-06-14

**Authors:** Chang-Ping Feng, Fang Wei, Kai-Yin Sun, Yan Wang, Hong-Bo Lan, Hong-Jing Shang, Fa-Zhu Ding, Lu Bai, Jie Yang, Wei Yang

**Affiliations:** 1 Shandong Engineering Research Center for Additive Manufacturing, Qingdao University of Technology, Qingdao, 266520 People’s Republic of China; 2 State Key Laboratory of Polymer Materials Engineering, College of Polymer Science and Engineering, Sichuan University, Chengdu, 610065 People’s Republic of China; 3 Institute of Electrical Engineering, Chinese Academy of Sciences, Beijing, 100190 People’s Republic of China

**Keywords:** Thermal conductivity, Flexible thermally conductive films, Heat transfer mechanism, Interface thermal resistance, Thermal management applications

## Abstract

Effective thermal management is quite urgent for electronics owing to their ever-growing integration degree, operation frequency and power density, and the main strategy of thermal management is to remove excess energy from electronics to outside by thermal conductive materials. Compared to the conventional thermal management materials, flexible thermally conductive films with high in-plane thermal conductivity, as emerging candidates, have aroused greater interest in the last decade, which show great potential in thermal management applications of next-generation devices. However, a comprehensive review of flexible thermally conductive films is rarely reported. Thus, we review recent advances of both intrinsic polymer films and polymer-based composite films with ultrahigh in-plane thermal conductivity, with deep understandings of heat transfer mechanism, processing methods to enhance thermal conductivity, optimization strategies to reduce interface thermal resistance and their potential applications. Lastly, challenges and opportunities for the future development of flexible thermally conductive films are also discussed.

## Highlights

- The state-of-the-art progress of flexible thermally conductive films with ultrahigh in-plane isotropic thermal conductivity (*k*) and potential application are summarized.
- The heat transfer mechanism, processing methods to enhance *k*, optimization strategies to reduce interface thermal resistance of flexible thermally conductive films are reviewed.
- The limitations and opportunities for the future development of flexible thermally conductive films are proposed.

## Graphical abstract

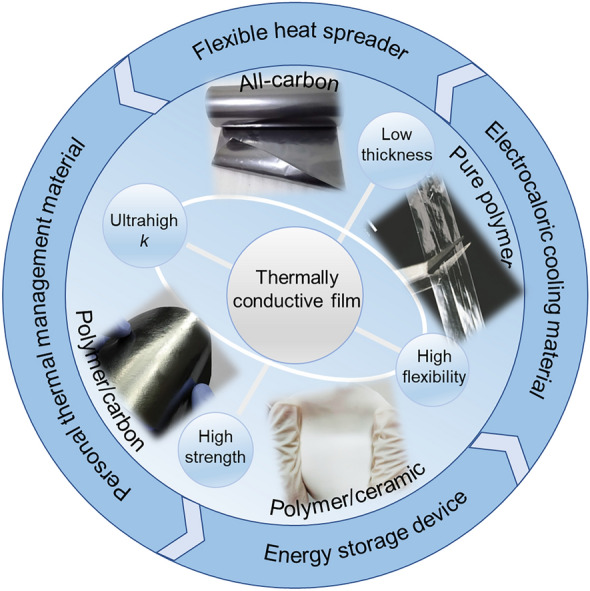

## Introduction

**Fig. 1:**
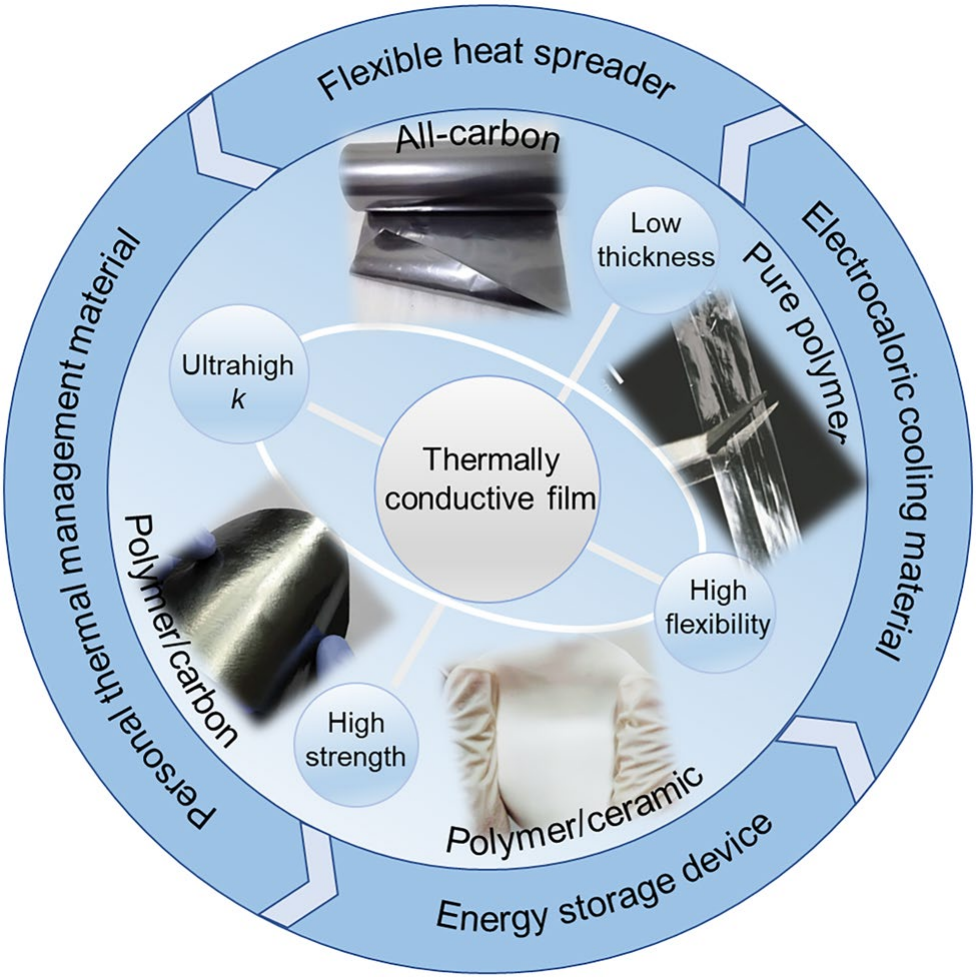
Overview of flexible thermally conductive films for thermal management

Owing to the rapid development of high-power densification and high miniaturization of electronics, effective thermal management is quite urgent for electronics in the fields of communication, military and energy storage systems, and the main objective of thermal management is to remove excess energy from electronics to ambient environment [1–6]. Typical thermally conductive materials including thermal greases, thermal adhesives, thermal pads and bulk polymer composites with high isotropic thermal conductivity (*k*) have been widely used as thermal interface materials (TIMs), heat sink and heat spreader in the field of electronics [7, 8]. However, thermally conductive films with excellent flexibility and superhigh in-plane *k* are getting more attention than conventional thermally conductive materials, and significant efforts have been made in developing flexible in-plane thermally conductive films including pure polymer films, all-carbon films and polymer-based composite films (Fig. 1) [9–13].

**Fig. 2:**
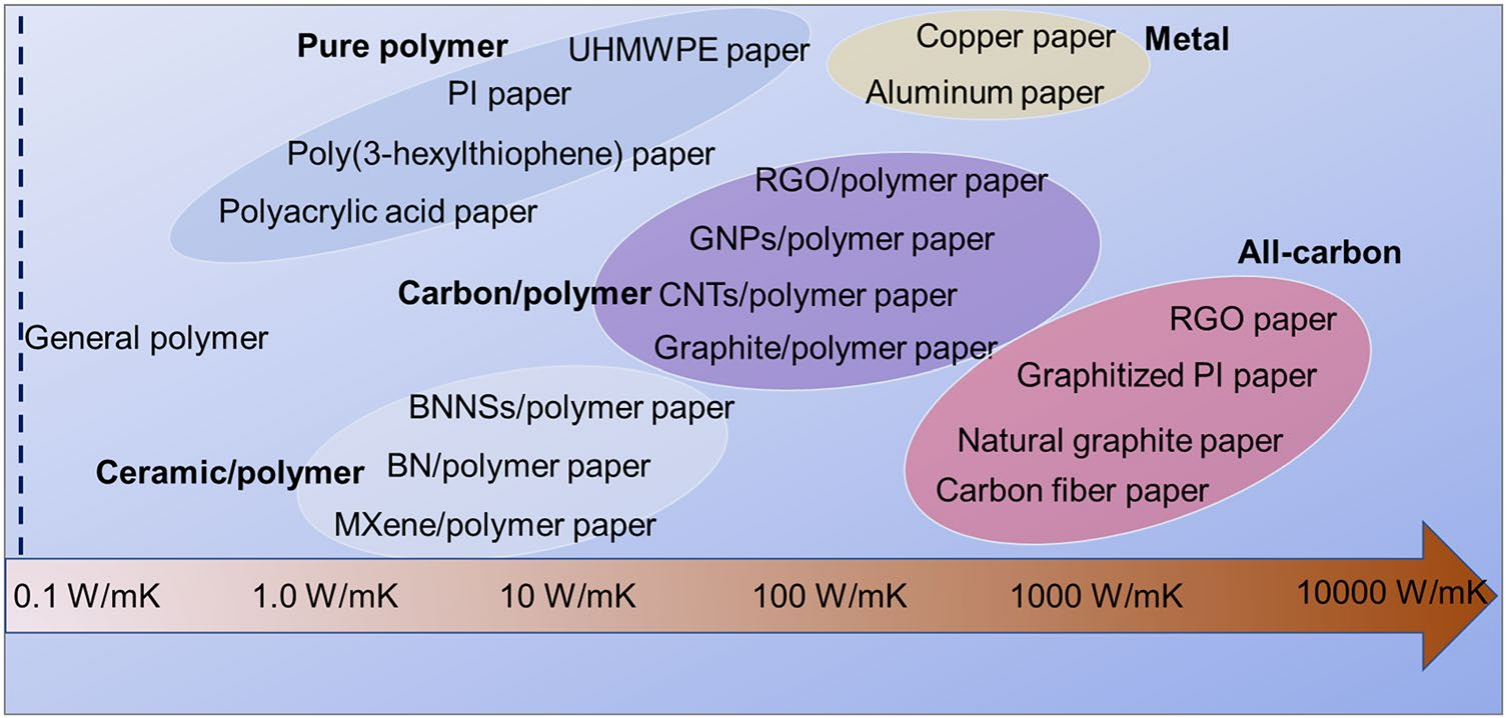
The summary of flexible thermally conductive films with different in-plane *k*

Recently, extensive research efforts have been dedicated to fabricating paper-like composites with hierarchical structures and ultrahigh in-plane *k*, such as reduced graphene oxide (RGO) film (1940 W (mK)^−1^ [14]), carbon nanotubes (CNTs) film (200 W (mK)^−1^ [15]), boron nitride /polyvinyl alcohol (BN/PVA) film (120.7 W (mK)^−1^ [16]), boron nitride nanosheets/poly(diallyl dimethyl ammonium chloride) (BNNS/PDDA) film (200 W (mK)^−1^ [17]) and BNNS/nanofibrillated cellulose (NFC) film (145.7 W (mK)^−1^ [18]). Constructing an effective thermally conductive filler network and reducing phonon scattering at the interfaces (filler/filler, polymer/polymer and filler/polymer) are the main strategies to obtain thermally conductive polymer composite films [19–21]. More impressively, ultradrawn ultrahigh molecular weight polyethylene (UHMWPE) films with metal-like *k* of 63 W (mK)^−1^ have been achieved [22], while the *k* of common polymer is just around 0.2 W (mK)^−1^. Transforming the molecular chain structure of polymers into regular arrangement in a certain direction during polymerization or ultradrawing processing is the main strategy to obtain intrinsically thermally conductive polymer films [23, 24]. In summary, thermally conductive films with excellent flexibility and ultrahigh *k* have been extensively studied. Figure 2 presents the summary of thermally conductive films with different in-plane *k*.

Unfortunately, most of these flexible thermally conductive films cannot be used in common thermal management applications such as TIMs to effectively transfer the heat from heat source to heat sink vertically, because these films usually showed very low through-plane *k* [25, 26]. However, compared to isotropic thermally conductive materials, these thermally conductive films with low thickness, high mechanical strength and excellent flexibility show great potential in thermal management applications such as flexible heat spreader, wearable technologies, personal thermal management, on-skin electronics and energy storage devices, which can effectively transfer the heat from high-temperature positions in the in-plane direction without the compromise of electronics flexibility, while holding back the effect of adjacent components [27, 28]. For example, owing to high in-plane *k* and adequate breathability, graphene/cellulose films can be used as heat management materials in on-skin electronics [29].

In the past few years, a number of significant progresses focusing on thermally conductive materials have been witnessed. However, a comprehensive review of flexible thermally conductive films involving heat transfer physics, thermal conductivity measurements, fabricating methods, optimization strategies to reduce interface thermal resistance (ITR) and their potential applications is rarely reported. Here, this review aims to summarize the state-of-the-art progress of flexible thermally conductive films with ultrahigh in-plane *k*. First, thermal transport mechanism in polymer and composites are discussed, and the generally used *k* measurement techniques of thermally conductive films are introduced. Then, we highlight the fabrication methods of emerging flexible high-performance thermally conductive films including pure polymer films, all-carbon films, carbon/polymer composite films and ceramic/polymer composite films, and their potential thermal management applications. Finally, we put forward some views on the challenges and some key points for the exploration of high-performance flexible thermally conductive films.

## Heat Transfer and Thermal Conductivity Measurements of Thermally Conductive Films

### Heat Transfer of Polymers

In crystalline solids, the thermal transport can be understood as the propagation of phonons and their scatterings, and the *k* of solid materials can be generally written as an integration over the phonon spectrum [23, 30]:

1 $$k = 1/3\smallint \hbar \omega N\left( \omega \right)\frac{\partial f}{{\partial T}}v^{2} \left( \omega \right)\tau \left( \omega \right)d\omega = 1/3\smallint C\left( \omega \right)v^{2} \left( \omega \right)\tau \left( \omega \right)d\omega$$

where *ν* is the phonon group velocity, *C(ω)* is the spectral specific heat, *N(ω)* is the density of state, *∫dω* is integrating over the entire phonon spectrum and summing over all polarizations and *f* is the Bose–Einstein distribution [23, 30].

**Fig. 3:**
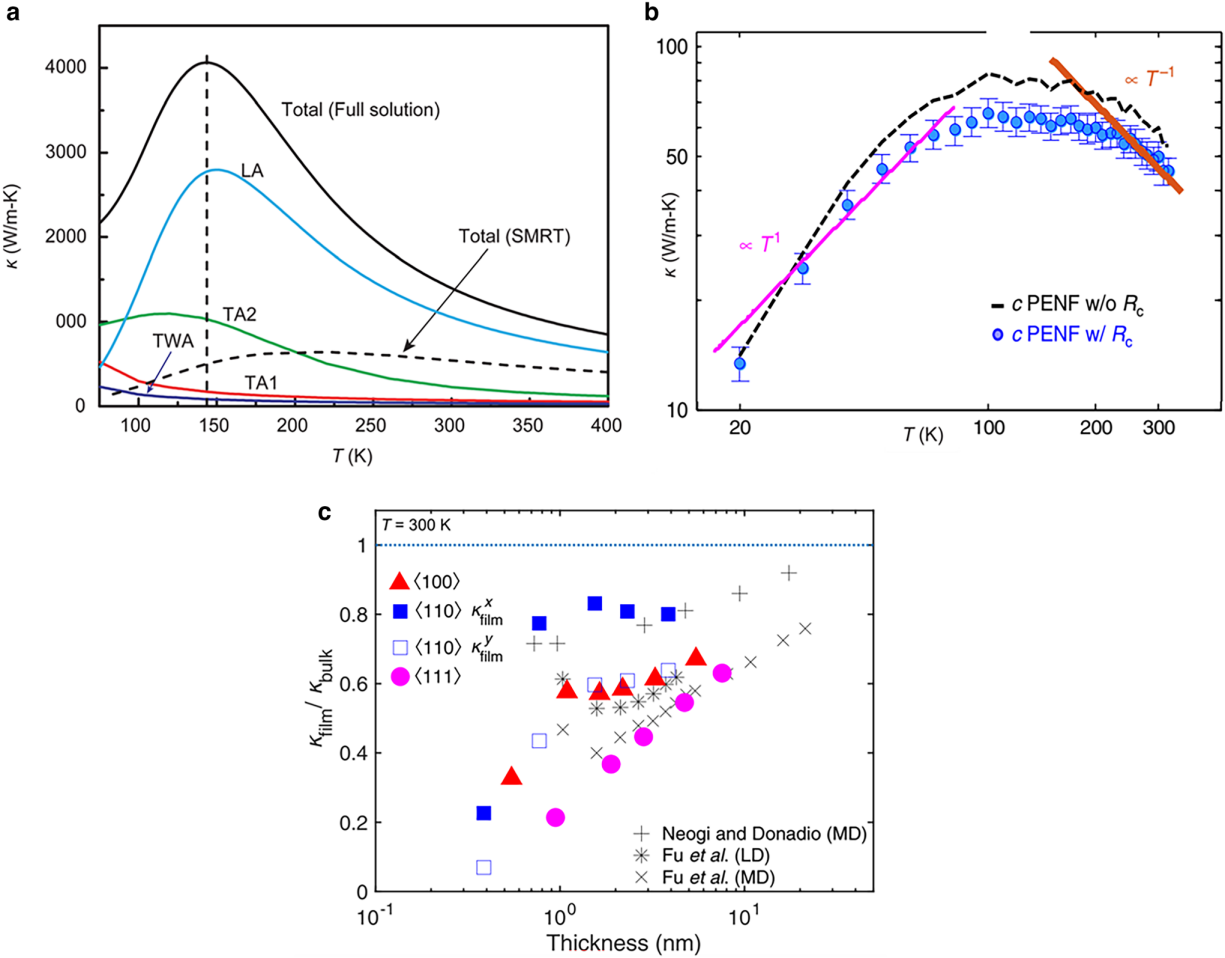
**a** Variations of the axial *k* of an infinite one-dimensional PE chain as a function of temperature, along with the absolute *k* contributions from the four acoustic phonon modes and the *k* values from the single-mode relaxation-time model. TA1 and TA2 are the two transverse phonon modes vibrating perpendicular to the chain axis, TWA is the twisting mode and LA is the longitudinal mode. Reproduced with permission from Ref. [31]. Copyright 2017, American Chemical Society. **b** *k* of PE nanofiber as function of temperature. Reproduced with permission from Ref. [3]. Copyright 2018, Springer Nature. **c** Ratio of *k* of thin PE films to *k* of PE bulks as function of thickness dependence. Reproduced with permission from Ref. [33]. Copyright 2021, American Physical Society

The phonon transfer mechanism of crystalline polymers is more complicated than crystalline solids, because crystalline polymers possess complicated multilevel structure. To understand the heat transfer mechanism in polymers, the first-principle and molecular dynamics (MD) simulation have been utilized to study the heat conduction in polymer chains and single crystals. In particular, Huang et al. [31] used the first-principle-base anharmonic lattice dynamics to investigate the *k* of both bulk and single-chain polyethylene (PE), and a very high *k* of 1400 W (mK)^−1^ in single-chain PE was observed compared with 237 W (mK)^−1^ for bulk PE crystals. Figure 3a shows the phonon transfer contributions from each phonon branch, showing that longitudinal phonon modes dominate the thermal transport in PE chains, while transverse phonon branches with quadratic dispersions contribute little to *k* due to their vanishing group velocities and limited lifetimes in the long wavelength limit. Theoretically, the *k* of crystalline polymers along the chain alignment direction can be estimated with Eq. (2) [30, 32]:

2 $$k = \frac{{213E^{3/2} \delta }}{{M_{b}^{0.48} M_{s}^{0.02} P^{3} V^{2/3} T}}$$

where *E* is the averaged bond energy in the backbone of crystalline polymers, $\delta$ is the in-plane bond ratio, *M*_*b*_ is the averaged mass of the backbone atoms, *M*_*s*_ is the average atomic mass of side chain atoms, *P* is the chain rotation ratio, *T* is the temperature and *V* is the total volume of the unit cell [30]. The model can be readily applied to calculate *k* of crystalline polymers without complex simulations. From the model, it can be concluded that strong bond in backbone, small side chain mass, small unit cell and aligned backbone within one plane are preferred to obtain high *k* [32]. Using MD simulation, Luo et al. [34] also found that π-conjugated polymers with aligned and rigid backbones showed higher *k*, due to large phonon group velocities and suppressed segmental rotations which enabled long phonon mean free paths.

The *k* of polymers shows strong morphology dependence. For example, ultradrawn process can significantly increase the crystal and chain arrangement, giving rise to a high intrinsic *k* in the alignment direction. The observed increase of *k* in oriented crystalline polymer materials can be ascribed to the crystal orientation for draw ratios lower than 80, followed by the increase in crystal size at higher stretching [22]. In semicrystalline polymers such as polyethylene oxide (PEO), the alignment of polymer chains and the orientation of crystalline grains usually exist simultaneously. In this case, Lu et al. [35] found that the polymer chain alignment in the amorphous region showed primary contribution for thermal transfer enhancement.

Besides, the phase transition of polymers can destroy the along-chain segmental order, which leads to a significant reduction of *k* by almost one order of magnitude [34]. For example, when the polymer gets into rubbery state, the molecular chains of the amorphous phase begin to move, leading to a great reduction in the phonon mean free path and *k* [36]. Therefore, the *k* of polymers shows a strong temperature dependence. Figure 3b gives the *k* of PE nanofiber as function of temperature. It can be seen that the *k* increases as T^1^ over the low-temperature range and can reach to 90 W (mK)^−1^ owing to the increased heat capacity and then reduces as T^−1^ at higher temperature owing to anharmonic phonon scattering in the crystals [3]. Bai et al. [36] studied the effect of temperature on the *k* of poly(L-lactic-acid) (PLLA) material, and the results suggested that the *k* of PLLA increases with the increasing of temperature when the temperature is below the glass transition temperature, but the increase is very limited.

Owing to the disordered nature of amorphous polymers, the thermal conduction of amorphous polymer is inherently complex compared with crystalline polymers. Meanwhile, the understanding of their thermal transport mechanism is relatively poor. Many thermal transport mechanisms in crystalline solid cannot be used to illuminate the thermal transfer in amorphous polymers. The *k* of bulk amorphous polymers is inhibited by highly coiled and entangled intrachain structure, loose chain packing with voids dampening the speed at which vibrations propagate, and weak nonbonding interchain interactions (e.g., van der Waals force) [37]. The minimum thermal conductivity model (MTCM) can be used to well describe the *k* of amorphous polymers [38], and the minimum *k* based on MTCM is expressed as:

3 $$k_{{{\text{min}}}} = \left( {\frac{\pi }{6}} \right)^{1/3} k_{B} n^{2/3} \mathop \sum \limits_{i = 1}^{3} V_{i} \left( {\frac{T}{{{\Theta }_{i} }}} \right)^{2} \mathop \int \limits_{0}^{{{\Theta }_{i} /T}} \frac{{x^{3} e^{x} }}{{\left( {e^{x} - 1} \right)^{2} }}{\text{d}}x$$

where *n* is the atomic density, ${\Theta }_{i}$ is the Debye cut-off temperature, *k*_*B*_ is the Boltzmann constant and *ν*_*i*_ is speed of sound [39]. Hsieh et al. [40] measured the pressure dependence of the *k* of poly(methyl methacrylate) through time-domain thermoreflectance and silicon carbide anvil-cell techniques and demonstrated that the *k* can be accurately predicted by MTCM.

Weak chain interactions have been considered to be a bottleneck for enhancing *k* of polymers, thus, enhancing chain interaction and introducing covalent crosslinkers between polymer chains have been an important route for enhancing *k* [41]. For example, Kim et al. [42] systematically explored the effect of several different H-bonding strategies, and the results demonstrated that given blends of poly(N-acryloyl piperidine)/poly(acrylic acid) with an exceptionally homogeneous distribution of strong H-bonds showed the highest *k* of 1.5 W (mK)^−1^. Pipe et al. [41] studied the mechanism of heat transfer in crosslinked polymers, and found that crosslinker can effectively enhance *k* when crosslinker is short in length and can bring polymer chains closer to each other, and the interchain interactions were highly dependent on polymer chains distance.

Low dimensionality of polymers can effectively enhance the *k*, due to the reduction of scattering channels and the removal of inter-layer coupling governed by anharmonic van der Waals force [31]. Thus, nanoscale polymer films or fiber show much higher *k* than bulk polymers. However, as the thickness of films is comparable to or smaller than that of phonon wavelength, the phonon transport properties will be significantly changed (Fig. 3c) [33]. The anharmonic lattice dynamics calculations suggest that anharmonic coupling between surface and internal phonons localized in thin films significantly suppresses the overall in-plane thermal conduction, and surface-internal phonon scattering predominantly affects the reduced *k* of thin films [33].

### Heat Transfer of Composites

The heat transport mechanism in composites is more complex than that in pure polymers, and the *k* of composites depends on multiple factors including thermally conductive filler characteristics (size, shape, crystal structure) [43–47], polymer matrix characteristics (crystallization degree, molecule weight, interchain interaction and chain orientation) [3, 24, 36, 48], microstructure control of fillers (filler orientation and network structure) [49–51] and ITR (filler/filler, polymer/polymer, and filler/polymer) [52]. The parameters such as loading and intrinsic *k* of thermally conductive filler play a decisive role in the resultant *k* of composites. When filler loading is low, thermally conductive fillers are challenging to construct effective thermally conductive pathways to transfer the phonon. Thus, high loading and high intrinsic *k* of fillers are needed to improve the *k* of composites significantly. When the filler is identified, microstructure control of filler has become a more crucial factor in constructing effective thermal transport pathways, reducing interfacial thermal resistance between filler/polymer and obtaining higher *k* [53]. For example, the perfect alignment of RGO platelets undoubtedly endows the RGO films with extraordinary in-plane* k *owing to the intrinsically high intrinsic *k* of graphene along the basal planes and the limited filler/polymer interfaces [54]. The detailed explanations of the relationship between the *k* of composites and multiple factors have been systematically summarized by some reported reviews [53, 55–57].

Theoretical simulation of thermally conductive composites can accurately predict the *k* of composites, quantitatively analyze the influence of various factors on *k* and describe the complex heat transfer mechanisms in composites. At the microscopic level, the common method of theoretical simulation of composites is the molecular dynamics methods, including equilibrium molecular dynamics and non-equilibrium molecular dynamics [43]. The non-equilibrium molecular dynamics method is more commonly used for the *k* calculation of composites because this method can account for arbitrary shapes and structures of the composites without any assumptions or simplifications and can describe the vibrational motion of phonons in detail [43]. For example, Sun et al. [44] used non-equilibrium molecular dynamics simulation to study the effects of microstructure control of fillers, thermal property mismatch at the interface and polydispersity of fillers on the *k* values of composites. The simulation results demonstrated that a larger mismatch at the interface between polymers and fillers leads to a smaller *k*. In addition, the fillers with a larger interfacial area perpendicular to heat flow or a more significant interface density also yield a smaller *k*. At the macroscopic level, the finite element method and the finite difference method are widely used methods to study the heat transfer mechanisms of composites. For example, Tsekmes et al. [45] used the finite element method to investigate the effect of filler shape, size, intrinsic *k*, the interaction between fillers, and surface modification on the *k* of composites. The simulation results demonstrated that fillers with a high aspect ratio could more easily construct thermally conductive pathways along the direction of the heat flow.

**Fig. 4:**
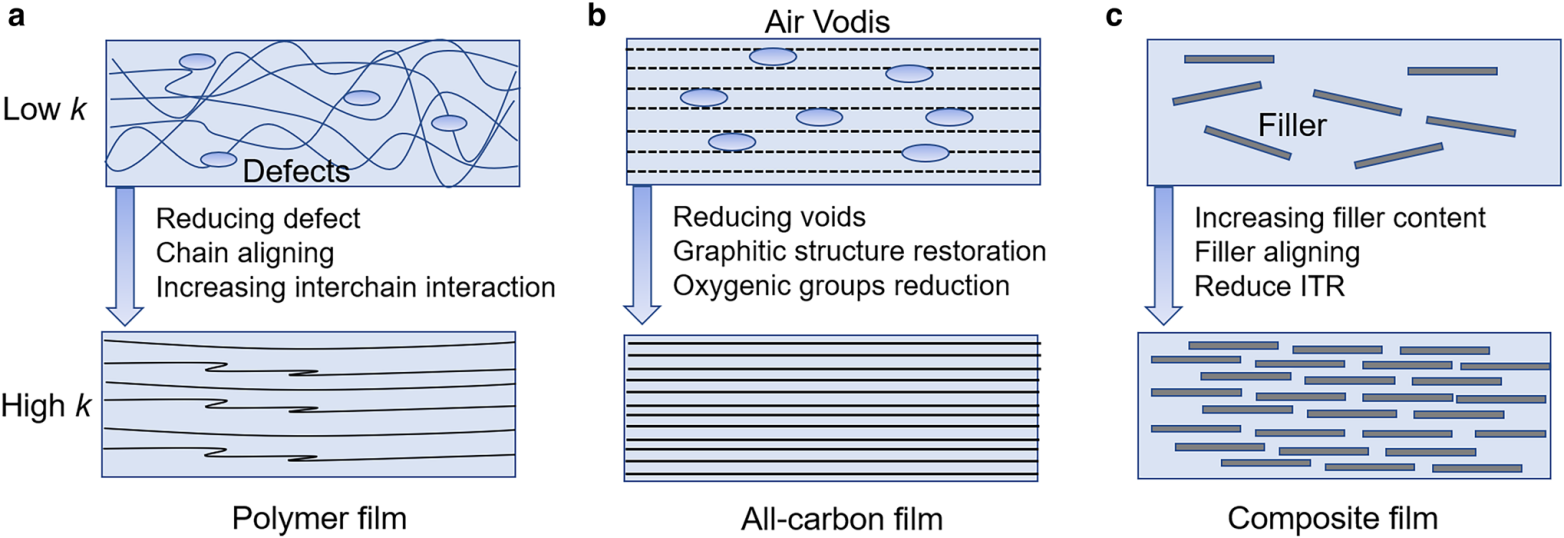
The construction of thermal transport pathways in different kinds of films: **a** polymer film, **b** all-carbon film, and **c** composite film

Although the percolation threshold phenomenon in thermally conductive epoxy/graphene composites have been reported in several publications [58, 59], unlike percolation threshold phenomenon in electrically conductive materials, most experimental studies have failed to observe the percolation threshold phenomenon in thermally conductive materials. A main reason is that the polymer matrix cannot be treated as a perfect thermal insulator, and thus, phonon transport can still carry on through the polymer matrix. Therefore, the construction of the thermally conductive pathways will not cause an intense change in the *k* [60]. Although percolation threshold phenomenon is not obvious, the concept of “thermal transport pathways” has been widely used to explain the enhancement of *k* of composites [61, 62]. Figure 4 shows the thermal transport pathways in different kinds of films including polymer film, all-carbon film and composite film. Effective thermal transport pathways can be constructed by aligning polymer chains, aligning two-dimensional filler sheets, and overlapping of fillers across in-plane direction in the matrix of composite films, respectively. Simple compounding of thermally conductive fillers and polymers generally leads to random distribution of fillers and increase of filler/polymer interfaces, resulting in high ITR and limited *k* [63]. Thus, constructing effective thermal transport pathways and reducing interfacial thermal resistance between filler/polymer are the main strategies to fabricate highly thermally conductive composite films [64].

### Thermal Conductivity Measurements of Thermally Conductive Films

Although many thermal technologies have been developed to test the *k* of materials, the in-plane *k* of films can only be measured by thermal bridge method, laser flash method, 3ω method, the steady-state infrared thermography, optothermal Raman spectroscopy and Völklein method.

**Fig. 5:**
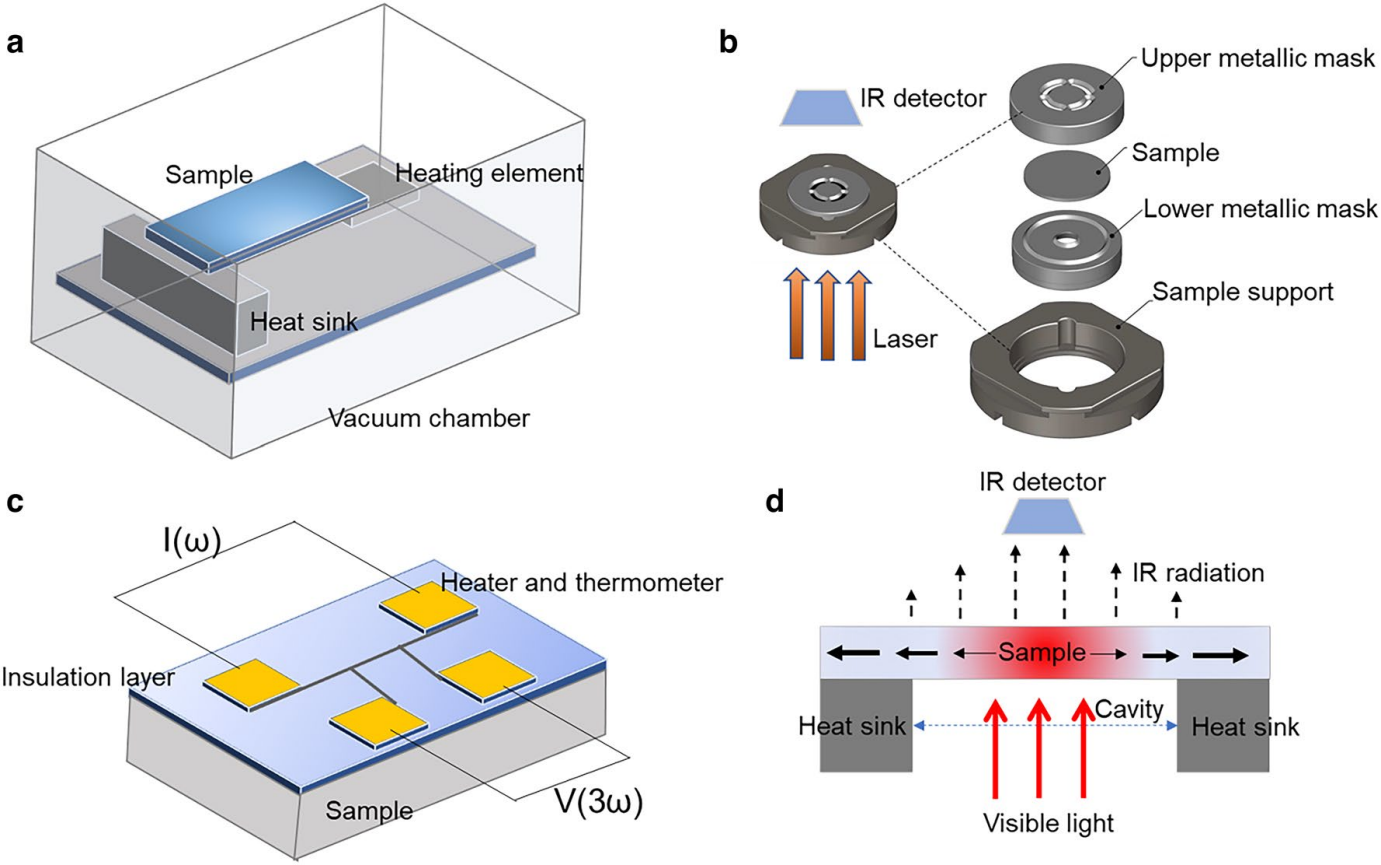
The widely used thermal conductivity measurements to test the in-plane *k* of films: **a** thermal bridge method, **b** flash method, **c** 3*ω* method, and **d** steady-state infrared thermography

Figure 5a shows the schematic of thermal bridge method for *k* measurement of films. The films sample is suspended between heater and heat sink inside a vacuum chamber, and the heat transfers from the heater to heat sink along the film sample. The in-plane *k* of films can be calculated as:

4 $$k = \frac{qL}{{{\text{Wd}}\left( {T_{1} - T_{2} } \right)}}$$

where *T*_*1*_*, T*_*2*_*, q, L, W and d* are the heater temperature, heat sink temperature, heat flux, sample length, width and thickness, respectively [65].

The laser flash method was first reported by Parker in 1960 [66] and is now the most popular technique to obtain in-plane *k* in scientific community. During testing a high-intensity light pulse is absorbed in the surface of samples, then the temperature of rear surface is tested and recorded by an infrared detector. The thermal diffusivity can be calculated as:

5 $$\alpha = \frac{1.388}{{\pi^{2} }}\frac{{d^{2} }}{{t_{1/2} }}$$

where *t*_1/2_ is the time for the rear surface to reach half of the maximum temperature rise. The *k* was calculated by $k=\alpha \times \rho \times {C}_{p}$, where *k, α, ρ* and *C*_*p*_ represent the thermal conductivity, thermal diffusivity, density and specific heat capacity of the composites, respectively. Before testing, both surfaces of sample should be coated with a thin graphite layer to improve the light absorption [67]. To test the in-plane *k* of films, film samples need to be held between two metallic masks, with the lower metallic mask possessing a circular hole in the central region to allow the light beam to pass and heat the central region of the sample (Fig. 5b). The upper metallic mask possesses arc-shaped openings through which the temperature can be monitored by an infrared detector. Lastly, a two-dimensional heat transport model is used to calculate the resultant in-plane *k* of film. The most commonly used commercial instruments is Netzsch LFA HyperFlash [68].

The 3*ω* method was first reported by Cahill in 1987 and has become commonly used to measure the *k* of polymer films and composite films [69]. As shown in Fig. 5c, when testing a thin metal strip is deposited on the top of the sample to serve as heater and thermometer, and an AC current at a frequency of *ω* is injected into the metal strip to create Joule heat at a frequency of *2ω*, and a *3ω* voltage signal can be detected by the lock-in amplifier [68]. The *k* can be determined by measuring the thermal response of the film-on-substrate system and comparing it with the thermal response in the absence of the thin film [70]. Ju et al. [71] have developed a 3ω method to test the in-plane *k* of films using a very narrow metal strip which is sensitive to the thermal conduction in both in-plane and through-plane direction.

Figure 5d shows the schematic of steady-state infrared thermography for *k* measurement of films. The thin film is suspended over a cavity in an opaque substrate using as heat sink and shadow mask for the illumination. The film is homogenously illuminated from below by visible light with an absorbed power density *Q*. A steady-state temperature gradient can be generated by homogeneous heating from absorbing visible light and heat dissipation from lateral heat sink [72]. The temperature distribution can be recorded by an infrared camera and fitted to the theoretical temperature distribution for the specific hole geometry to obtain the in-plane *k* of films [72]. The theoretical temperature distribution $T\left(r\right)$ can be given by Eq. (6):

6 $$T\left( r \right) = - \frac{Q}{{4{\text{kd}}}}(r^{2} - r_{0}^{2} ) + T_{0}$$

where *Q* is the absorbed power density, *k* is the in-plane thermal conductivity of the film, *r*_*0*_ is the radius of the cavity, *T*_*0*_ is the temperature of the substrate and *d* is the thickness of film [68].

Table 1 presents the comparison of the advantages, disadvantages and sample geometry of thermal technologies presented in the review to measure the in-plane *k* of films. In summary, various thermal technologies have been developed to test the in-plane *k* of films. However, a standardization agreement among the international scientific community on how to test in-plane *k* of films is urgently needed [73]. To ensure the accuracy and creditability of the results, some researchers prefer to crosscheck their results by different techniques [68].

**Table 1:** The comparison of advantages and disadvantages of thermal technologies presented in the review to measure the in-plane *k* of films [68, 73, 74]

Methods	Advantages	Disadvantages	Sample geometry
Thermal bridge method	Substrate effect eliminated; can obtain *k* directly	The effect of thermal radiation losses and contact thermal resistance is ignored; extensive microfabrication works	Nanoscale or microscale samples
Flash method	Wide temperature range; fast and noncontact; low cost	Need surface coating; need smooth film surfaces; need specific heat capacity and density	> 100 µm in thickness
3*ω* method	Can obtain *k* directly; insensitive to the radiation and convective heat losses	Microfabrication of the metal stripe; need good insulation layer	Sub-micrometer thick samples
Steady-state infrared thermography	Fast and noncontact; can obtain *k* directly	Sample needs substantial optical absorptance and large emissivity; need extensive calibrations of the experimental set up	Sub-micrometer thick samples

## High-performance Thermally Conductive Films

### Pure Polymer Films

Commonly, polymers are thermal insulators with low *k* of 0.02–0.2 W (mK)^−1^ owing to the very limited mean free path of heat-conducting phonons caused by defects, such as voids, polymer chain ends, entanglement, impurities and the amorphous arrangement of polymer chains [75]. Mixing with high-*k* filler is the most common method to enhance the *k* of polymers, but the introduction of high loading fillers inevitably leads to high cost, high density, poor mechanical properties and undesirable electrical and optical properties [37]. Thus, it is highly desired and urgently needed to develop pure polymers with high intrinsic *k*.

Recently reported pure polymer films with high intrinsic *k* are presented in Table 2. One can see that the most common molecular engineering strategies to enhance the intrinsic *k* of polymer includes the alignment of polymer chains through stretching, the increase of degree of crystallinity with heat treatment and the improvement in the molecules interactions [76, 77]. Atomistic simulations and numerical predictions suggested that individual crystalline PE chain exhibits extraordinary high intrinsic *k*, over 237 W (mK)^−1^ [31]. However, experimental validation of such a theoretically high *k* remains elusive [24].

**Table 2:** The summary of pure polymer films with high intrinsic *k*

Sample	Fabrication method	Strategy	*k* (W (mK)^−1^)	Measurement method	Refs.
UHMWPE films	Ultradrawing	Chain alignment	62	Home-built steady-state system	2019 [24]
UHMWPE films	Ultradrawing	Chain alignment, Enhancing crystallinity	63	Laser flash technique (LFT)	2017 [22]
UHMWPE films	Solution gel-shearing	Chain alignment	10.74	Hot disk	2021 [78]
Poly(3-hexylthiophene)	Bottom-up oxidative chemical vapor deposition	π-π stacking interactions	2.2	Time-domain thermoreflectance	2018 [79]
Grafted brushes of poly(3-methylthiophene)	Surface-initiated polymerization	Polymer brush alignment	2.0	Time-domain thermoreflectance	2016 [80]
Poly(*N*-acryloyl piperidine)/poly(acrylic acid)	Spin casting	Enhancing interchain H-bonds interactions	1.5	3*ω* method	2014 [42]
Polyacrylic acid	Spin casting	Enhancing degree of ionization	1.2	3*ω* method	2017 [81]
PLLA	Hot compression and heat treatment	Enhancing crystallinity	0.348	LFT	2018 [36]
Liquid crystalline polyimide (PI)		Retaining microscopically ordered chain structure	2.11	/	2021 [82]

**Fig. 6:**
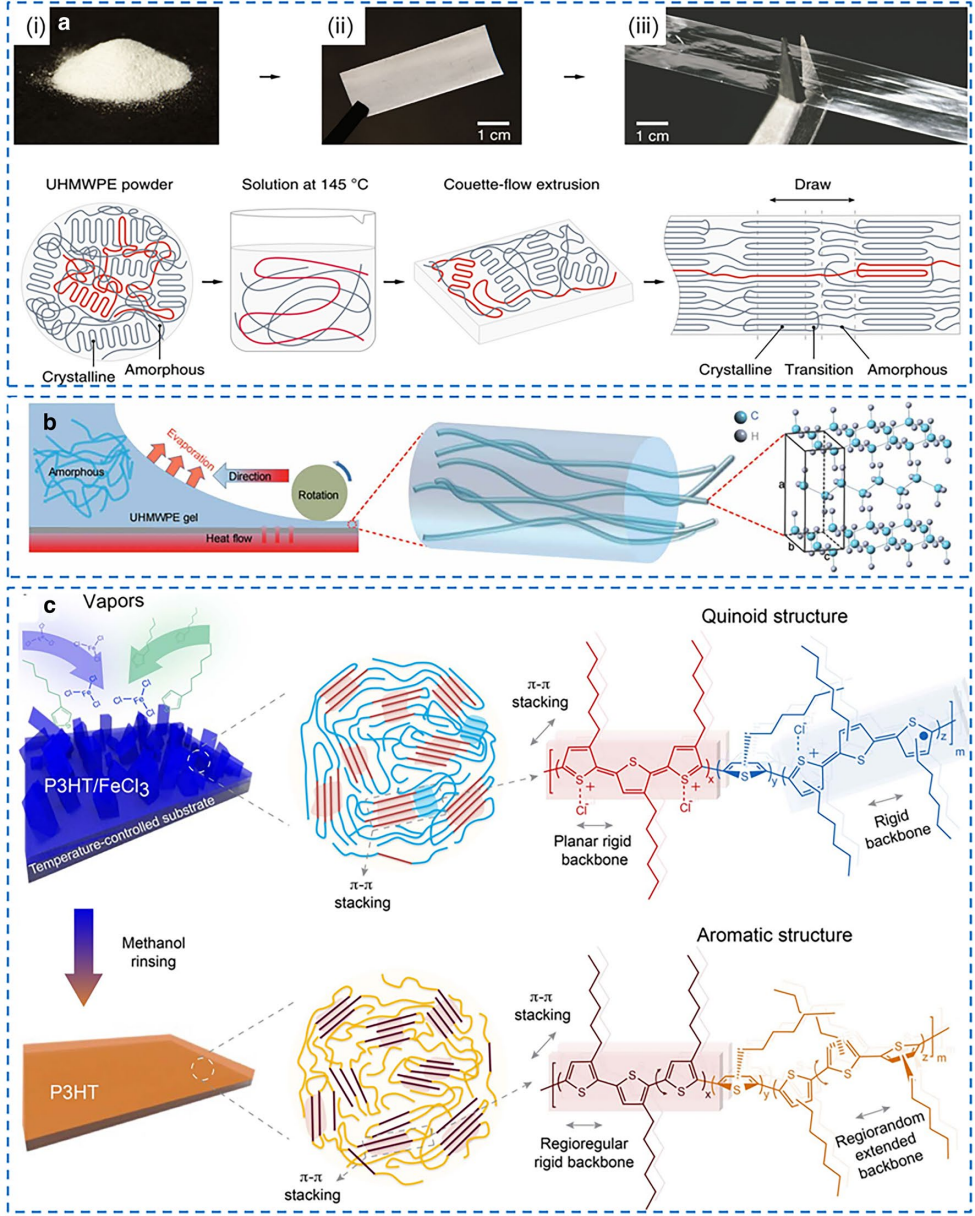
**a** Fabrication of UHMWPE films with high intrinsic *k* via ultradrawing process. Reproduced with permission from Ref. [24]. Copyright 2019, Springer Nature. **b** Fabrication of UHMWPE films with high intrinsic *k* via solution gel-shearing process. Reproduced with permission from Ref. [78]. Copyright 2021, American Association for the Advancement of Science. **c** Fabrication of conjugated poly(3-hexylthiophene) films with intrinsic *k* using oxidative chemical vapor deposition. Reproduced with permission from Ref. [83] Copyright 2018, American Association for the Advancement of Science

Notably, the *k* of ultradrawn PE nanofibers with diameters of 50–500 nm can reach up to 104 W (mK)^−1^, because the aligned polymer chains in ultrdrawn nanofibers can serve as efficient phonon transport pathways along the axis [76]. Also, pure PE-based polymer films with ultrahigh intrinsic *k* were fabricated by stretching or shearing. For example, Chen et al. [24] reported a ultrdrawn PE film with metal-like *k* of 62 W (mK)^−1^, and the thermal modeling indicates that the films consist of nanofibers with crystalline and amorphous regions, and the non-crystalline chain in the amorphous regions can achieve a high *k* via chain disentanglement and alignment (Fig. 6a). Similarly, Ronca et al. [22] fabricated PE films with the reduced number of entanglements by uniaxially and biaxially stretching in the solid-state without any solvent, and the increase of *k* showed two separate regimes which was attributed to the initial crystal orientation and subsequent chain extension. Recently, Ren et al. [78] reported a very simple solution gel-shearing method to fabricate PE films with an in-plane *k* of 10.74 W (mK)^−1^, which is ascribed to the alignment and close packing of ultrahigh-molecular weight crystalline chains, facilitating the formation of separated nanocapacitor with high *k* (Fig. 6b). In brief, a shearing rod drags the UHMWPE solution gel across a heated substrate while driving the gel solution between the rod and the substrate, and then the UHMWPE film is formed and peeled off from the glass substrate at room temperature [78]. The used solution gel-shearing process is more facile than widely used ultradrawing process. However, toxic solvent is inevitably used to dissolve UHMWPE in this method.

In addition to using stretching or shearing process to enhance the alignment of molecular chains, design specific monomer in polymerization process is another method to enhance the alignment of molecular chains. More specifically, Gu et al. [82] synthesized a high thermally conductive liquid crystalline PI films by optimizing the liquid crystal range to fit the curing temperature, and the molecular chains in PI films showed a perfectly ordered structure which can be maintained after solidification, thus giving the PI film a high intrinsic in-plane *k* of 2.11 W (mK)^−1^.

Besides, adjusting the molecules interactions is another effective route to enhance intrinsic *k* of pure polymers. As shown in Fig. 6c, Chen et al. [83] reported a conjugated poly(3-hexylthiophene) film prepared by oxidative chemical vapor deposition. By simultaneously harnessing the strong conjugated bonds along polymer chains and the π-π interactions between polymer chains, the films delivered an in-plane *k* of 2.2 W (mK)^−1^. Recently, Shanker et al. [81] used electrostatic repulsive forces between ionized pendant groups to stretch the polyelectrolyte backbone at the molecular level, resulting in strong ionic interchain interactions and stiffed polymer chain conformations which both contributed to significantly improved *k*. The *k* of polyacrylic acid films increased with increasing ionic interchain interactions, reaching up to 1.2 W (mK)^−1^.

To date, several kinds of pure polymer films with high intrinsic *k* have been successfully fabricated. However, it remains a long-standing challenge to enhance the *k* of polymers by simultaneously engineering the intramolecular and intermolecular interactions using more facial and effective methods, which is the key to realizing diverse applications in industrial thermal management. In addition, complicated fabrication process, widely used toxic solvents and high cost still hamper their large-scale production.

### All-Carbon Films

**Fig. 7:**
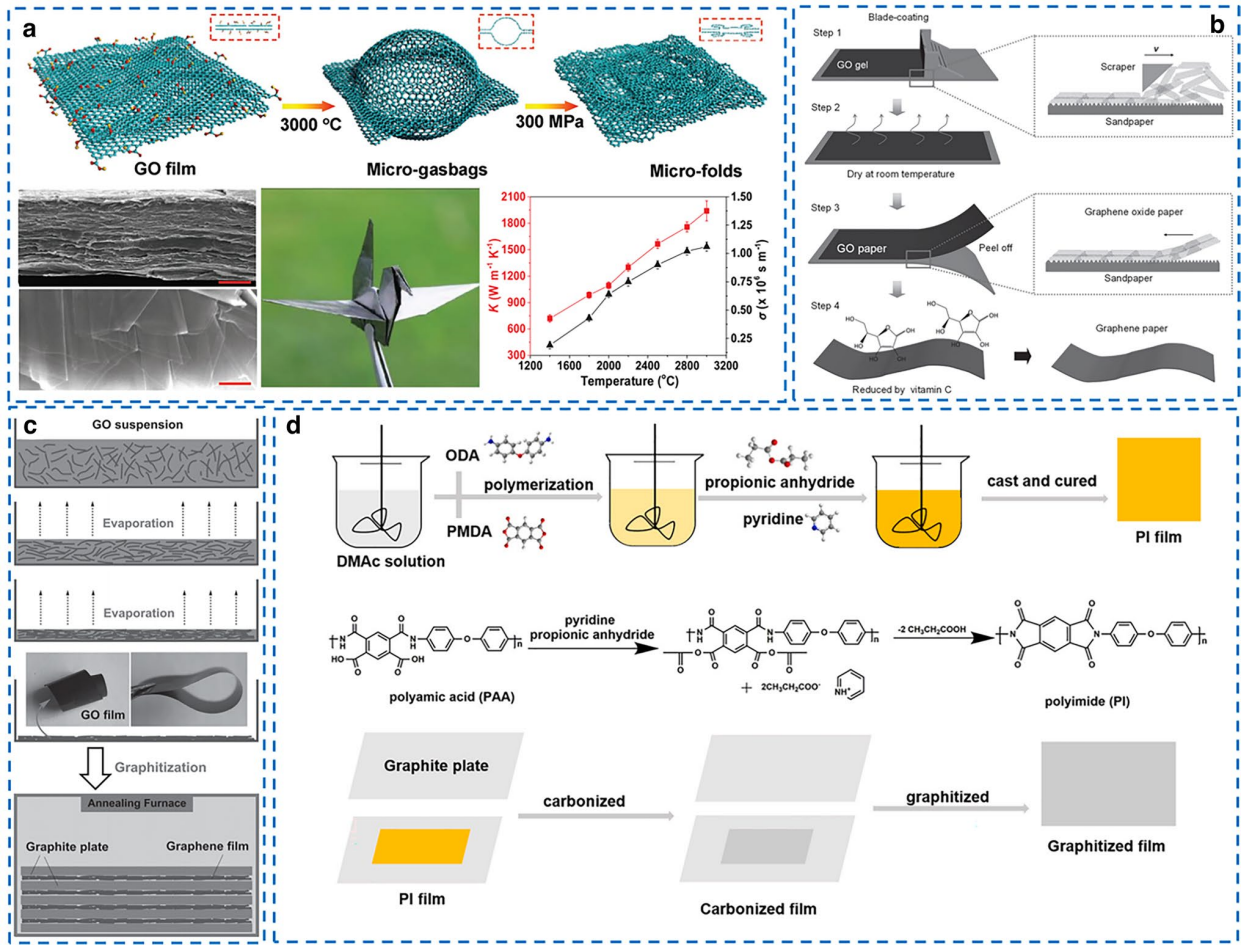
Fabrication of thermally conductive all-carbon films. **a** Preparation process of RGO film with micro-folds via high-temperature treatment and hot compression. Reproduced with permission from Ref. [14]. Copyright 2017, Wiley–VCH. **b** Preparation process of RGO film via blade coating and vitamin C reduction Reproduced with permission from Ref.[88]. Copyright 2017, Wiley–VCH. **c** Preparation process of RGO film by the evaporation of GO suspension and followed graphitization process. Reproduced with permission from Ref. [2] Copyright 2014, Wiley–VCH. **d** Preparation process of graphitized PI film by graphitization process of pure PI. Reproduced with permission from Ref. [102]. Copyright 2021, Elsevier

Owing to low atomic mass, simple crystal structure, low anharmonicity and strong bonding, graphene possesses a distinguished intrinsic *k* of 5300 W (mK)^−1^, thus graphene sheets have become a promising building block for constructing ultrahigh thermally conductive films [14, 84, 85]. Table 3 presents the summary of recently reported thermally conductive all-carbon films with high in-plane *k*. RGO films with *k* of 60–1940 W (mK)^−1^ [86] have been fabricated by thermal annealing of graphene oxide (GO) films at high temperatures over 1000 °C and hot compressing. More specifically, Gao et al. [14] used giant GO with an average lateral size of 108 μm as raw material at a high temperature of 3000 °C and ultrahigh pressure of 300 MPa to yield defect-free RGO film, and the resultant RGO film exhibited a promising *k* of 1940 W (mK)^−1^ and excellent flexibility (Fig. 7a). Noted that blade coating and solution casting are more facile than vacuum filtration to obtain GO film (Fig. 7b-c). The *k* of RGO film depends heavily on the graphitic structure restoration at high-temperature thermal annealing condition (2000–3000 °C) [52, 87, 88], which leads to high manufacturing cost and restricts their practical thermal management applications [89, 90].

**Table 3:** The summary of thermally conductive all-carbon films with high in-plane *k*

Sample	Fabrication method	In-plane *k* (W (mK)^−1^)	Measurement method	Flexibility and toughness	Refs.
RGO film	Solvent casting and thermal reduction	61	LFT	–	2015 [86]
RGO film	Solvent casting and thermal reduction	1100	LFT	–	2014 [2]
RGO film	Vacuum filtration and thermal reduction	1043.5	LFT	–	2014 [91]
RGO film	Electro-spray deposition and thermal reduction	1238.3	Self-heating method	–	2014 [92]
RGO film	Vacuum filtration and thermal reduction	1529	Self-heating method	–	2017 [93]
RGO film	Vacuum filtration and thermal reduction	1940	LFT	Elongation at break of 16%; 100,000 bending cycles	2017 [14]
RGO film	Vacuum filtration and thermal reduction	1390	LFT	Elongation at break of 2.2%	2015 [94]
RGO film	Vacuum filtration and thermal reduction	1300	LFT	Elongation at break of 4.65%; 1000 bending cycles	2018 [90]
RGO film	Blade coating and thermal reduction	739.56	LFT	–	2020 [95]
RGO film	Self-fusion strategy and thermal reduction	1224	LFT	–	2020 [96]
RGO film	Wet spinning and chemical reduction	1102.6	LFT	–	2020 [54]
RGO film	3D printing and thermal reduction	118.7	Self-heating-sensing electrothermal	–	2019 [97]
RGO film	LBL self-assembly	2514	Ramon method	–	2021 [98]
CNTs film	In situ alignment and further stretching-pressing	700.15	LFT	Elongation at break of 6.0%	2021 [99]
Graphene films	CVD and LBL assembly	2292	LFT	Elongation at break of 2.95%	2019 [100]
GPI	Polyimide graphitization	1781	LFT	–	2021 [101]
GPI	Polyimide graphitization	1092	LFT	–	2021 [102]
RGO/CF	Vacuum filtration	977	LFT	Elongation at break of 1.6%; 6000 bending cycles	2014 [103]
RGO/CNR	Vacuum filtration	890	/	–	2019 [104]
EGF film	Ball milling and vacuum filtration	242	LFT	Elongation at break of 0.5%	2020 [105]
RGO/EGF film	Ball milling and vacuum filtration	212	LFT	Elongation at break of 1.8%	2020 [106]

Thus, in order to reduce the thermal annealing temperature, Li et al. [90] adopted glucose as an activated carbon resource to repair the defects in graphene sheets at relatively a low temperature of 1000 °C for thermal treatment, and the *k* of resultant RGO films can also reach up to 1300 W (mK)^−1^. Apart from the high-temperature thermal annealing, chemical reduction can also be used to prepare RGO films. For example, Li et al. [54] adopted chemical reduction method employing hydroiodic acid as the reducing agent to reduce GO films, and the *k* of obtained RGO films can reach up to 1102.6 W (mK)^−1^. It is worth noting that the polymer residues on the surface of graphene films have a significant impact on the final *k*. For example, Kubal et al. [107] achieved effective modulation of in-plane *k* of graphene films in the range of 30% to 50% of the maximum value by controlling the polymer residue level and cluster separation. Their study demonstrated that the change in *k* is attributed primarily to the suppression of the out-of-plane acoustic (ZA) phonon transfer, which dominates the thermal transport in single-layer graphene at room temperature.

The chemical vapor deposition (CVD) method is more effective to obtain perfect graphene films, and Ruoff et al. [100] reported a graphene film with in-plane *k* of 2292 W (mK)^−1^ and in-plane electrical conductivity of 2.2 × 10^5^ S m^−1^ through stacking 100 layers of CVD-grown graphene. The outstanding performance of graphene films were attributed to the near-perfect in-plane crystalline order and unique stacking configuration in through-plane direction.

Moreover, natural graphite (NG) films (100–700 W (mK)^−1^) and graphitized polyimide (GPI) films (1000 [103] and 1750 W (mK)^−1^ [2]) with ultrahigh *k* have been produced from expanding graphite via compressing and graphitization of PI films at high temperatures, respectively [108]. Some polymers with high carbon yield as precursors can also be transformed into graphite structure after carbonization, a telling example is PI with dianhydride and diamine structures [101, 108]. Under high temperature, the elements of N, H and O can escape in the form of gas from PI films [108]. As shown in Fig. 7d, Min et al. [102] developed a GPI film with high in-plane *k* of 1092 W (mK)^−1^ after the graphitization of PI and found that 3.5 wt% catalyst pyridine can effectively improve the crystallinity and promote molecular arrangement and crystal lattice growth. In addition, some other thermally conductive all-carbon films, such as exfoliated graphene fluoride (EGF) films [105], RGO/EGF hybrid films [106], RGO/carbon fiber (CF) film [103], CNT film [99] and carbon nanoring(CNR)/RGO hybrid films [104], have also been developed by vacuum filtration, but they show lower *k* values than RGO films.

### Carbon/Polymer Composite Films

As mentioned above, all-carbon films with promising in-plane *k* have developed in the recent years, but they usually exhibit brittle and stiff features, which overshadow their industrial-scale applications [64, 109, 110]. Therefore, polymer component is generally introduced to enhance the toughness and flexibility of carbon films, such as PVA, natural rubber, silicone rubber, NFC and cellulose nanocrystals [111]. Among those polymer components, NFC, an eco-friendly and biodegradable polymer extracted from abundant natural cellulose resources [112, 113] shows more promising potential as surfactant to enable the favorable dispersion of fillers in aqueous solution and additive to improve the flexibility of the resulted films. However, the poor thermal resistance, complicated fabrication process and high cost of NFC are detrimental to its applications. Considering practical application requirements, PI and silicone rubber matrix with excellent mechanical property, high-temperature resistance and low dielectric content are more enticing candidates for commercial applications [114].

**Fig. 8:**
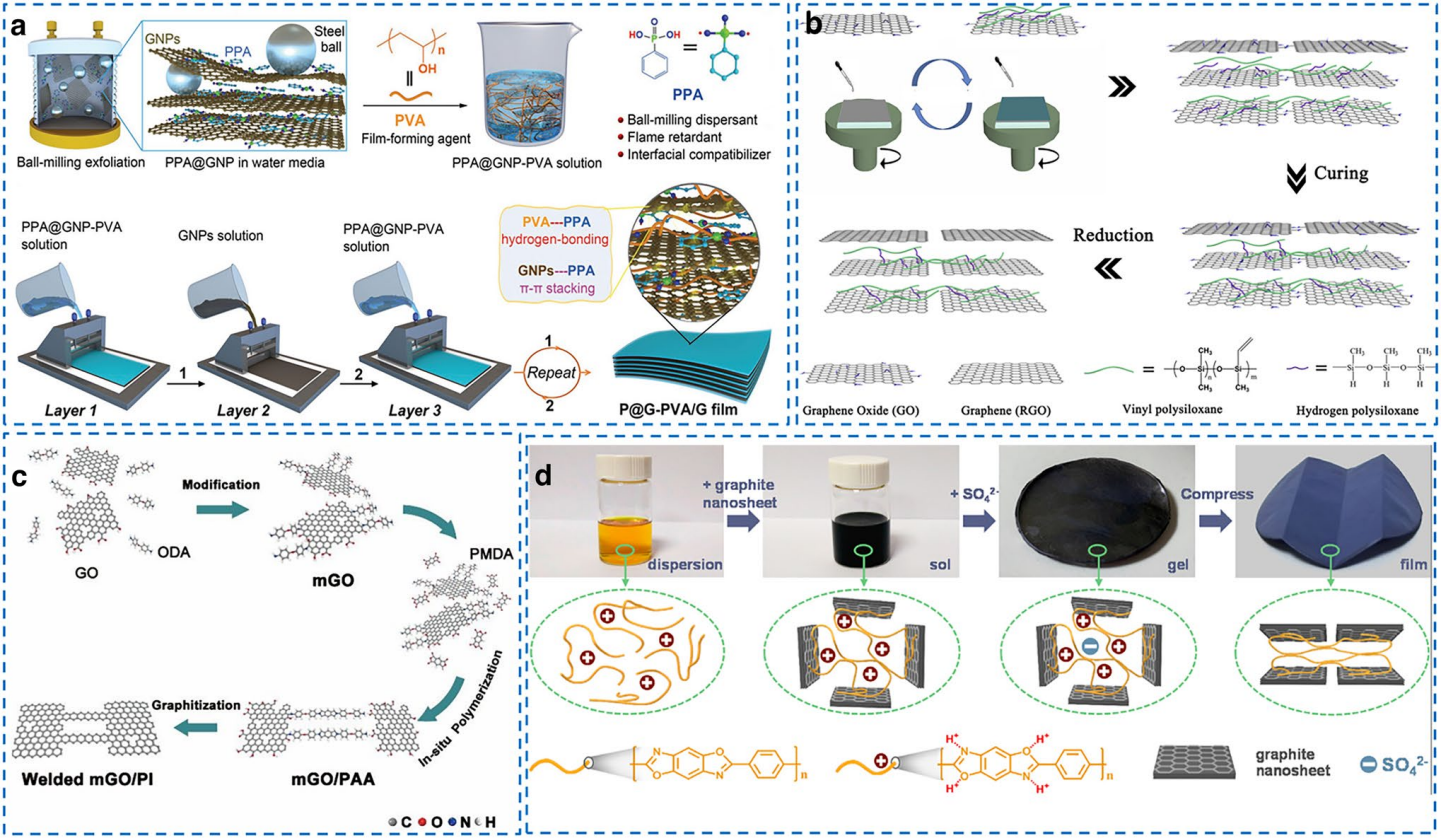
Fabrication of thermally conductive carbon/polymer composite films with high in-plane *k*. **a** Fabrication of PPA@GNPs/PVA films using LBL assembly approach. Reproduced with permission from Ref. [115]. Copyright 2021, Wiley–VCH. **b** Fabrication of silicone rubber/RGO films using spin-assisted LBL assembly method Reproduced with permission from Ref. [116]. Copyright 2018, Elsevier. **c** Fabrication of RGO/PI films using “modified welding” method. Reproduced with permission from Ref. [125]. Copyright 2019, American Physical Society. **d** Fabrication of poly(p-phenylene-2,6-benzobisoxazole) nanofiber/graphite film using a sol–gel film transformation approach. Reproduced with permission from Ref. [127]. Copyright 2021, Elsevier

Table 4 shows the summary of thermally conductive carbon/polymer composite films with high in-plane *k*. One can see that the introduction of polymer greatly reduces the *k* of all-carbon films, and the in-plane *k* of most of carbon/polymer-based films is in the range of 5–300 W (mK)^−1^. For example, as shown in Fig. 8a, Song et al. [115] reported a phenylphosphonic acid @GNPs (PPA@GNPs)/PVA film using modified GNPs and PVA multilayered films fabricated by a LBL assembly approach, and the resultant films exhibited a high in-plane *k* of 82.4 W (mK)^−1^, high flexibility and high tensile strength of 259 MPa. PPA was chosen as a surface modifier for GNPs filler to enhance the hydrophilicity and fire retardance. Similarly, in Fig. 8b, Zhang et al. [116] used spin-assisted LBL assembly method to fabricate RGO/silicone rubber films with an in-plane *k* of 2.03 W (mK)^−1^. The films with excellent mechanical flexibility and high stretchability can return to the original shape after unloading and be twisted to any shape. Recently, Wu et al. [117] fabricated a fluorinated graphene/PVA film with 93 wt% fluorinated graphene, and the in-plane *k* of films can reach up to 61.3 W (mK)^−1^. In summary, the introduction of polymer component into all-carbon films can effectively enhance the flexibility, but inevitably destroy the thermal transport pathways constructed by aligning two-dimensional carbon fillers and greatly reduce the *k*.

**Table 4:** The summary of thermally conductive carbon/polymer composite films with high in-plane *k*

Filler	Matrix	Fabrication method	Filler loading	In-plane *k* (W (mK)^−1^)	Measurement method	Flexibility and toughness	Refs.
RGO/nanodiamond	NFC	Vacuum filtration	10 wt%	14.35	LFT	Elongation at break of 4.26%	2020 [118]
RGO	NFC	LBL assembly	1 wt%	12.6	LFT	Elongation at break of 1.5%; 500 bending cycles	2017 [119]
Silver nanoparticles (AgNPs)@RGO	NFC	Vacuum filtration	9.6wt%	27.55	LFT	–	2020 [120]
AgNPs@GNPs	PVA	Solution casting and uniaxially stretching	10 wt%	8.45	LFT	–	2020 [121]
Fluorinated graphene	PVA	Vacuum filtration	93 wt%	61.3	LFT	Elongation at break of 5.7%	2019 [117]
RGO	PVA	Solution casting	5 wt%	4.9	LFT	Withstanding folding different shapes; Elongation at break of 180%	2021 [122]
MgO@RGO	NFC	Vacuum filtration and hot compression	20wt%	7.45	LFT	–	2020 [123]
GNPs	NFC	Vacuum filtration	75wt%	59.46	LFT	Elongation at break of 0.7%	2017 [124]
Fluorinated CNTs	NFC	Vacuum filtration	35 wt%	14.1	LFT	withstanding folding deformations; Elongation at break of 5.2%	2018 [112]
RGO	Silicon rubber	Spin-assisted LBL	2.53 wt%	2.03	LFT	Elongation at break of 325%	2018 [116]
RGO	PI	Solution casting	93 wt%	1352	LFT	2000 bending cycles	2019 [125]
RGO@carbon nitride	PI	Solution casting	10 wt%	6.08	LFT	Withstanding folding deformations	2021 [126]
graphite	Poly(p-phenylene-2,6-benzobisoxazole) nanofiber	Solution casting	90 wt%	179.8	LFT	Elongation at break of 24.3%; 10,000 bending cycles; toughness of 38.5 MJ/m^3^	2021 [127]
RGO	NR	Solution casting	38.54 vol%	20.84	LFT	10,000 bending cycles	2019 [128]

To simultaneously enhance the flexibility and *k* of carbon/polymer composite films, Yang et al. [125] developed a “modified welding” method to fabricate RGO/PI films, as shown in Fig. 8c. First, the GO sheets were modified by PI precursors via a grafting-to strategy to deliver active sites for further in situ polymerization of poly amic acid (PAA). After graphitization and imidization, PAA was converted to PI on the surface of RGO sheets, and the in-plane *k* of resultant RGO/PI films can reach up to 1352 W (mK)^−1^, 92.3% higher than that of pristine RGO films. Moreover, in Fig. 8d, Wang et al. [127] used poly(p-phenylene-2,6-benzobisoxazole) nanofiber to modify graphite film and found that the aligned graphite and robust 3D nanofiber network not only endows the modified graphite film with excellent mechanical property, but also leads to a promising in-plane *k* of 179.8 W (mK)^−1^. These reports provided feasible routes to improve the toughness of films and keep their excellent thermal conduction property simultaneously and demonstrated that maintaining perfect carbon filler pathway and constructing flexible polymer network is the key to obtain high-performance carbon/polymer composite films.

### Ceramic/Polymer Composite Films

In addition to carbon-based fillers, owing to the high intrinsic *k* of 220–420 W (mK)^−1^ [129], excellent electrical insulation, structure stability and antioxidant performance, BN has been a highly valued thermally conductive filler for the fabrication of thermal management materials [130]. Akin to NG, BN presents layered sp^2^-bonded hexagonally packed sheet structure. However, the valence electrons in NG are set in a conjugated system, which makes it an electron conductor, while the presence of two atoms with different electronegativities (2.04 for boron and 3.04 for nitrogen) endows BN partial ionic character, conferring to electron insulating properties [131]. These features make BN-based fillers superb candidates for thermally conductive materials in many industrial occasions where electrical insulation is required [132, 133].

**Fig. 9:**
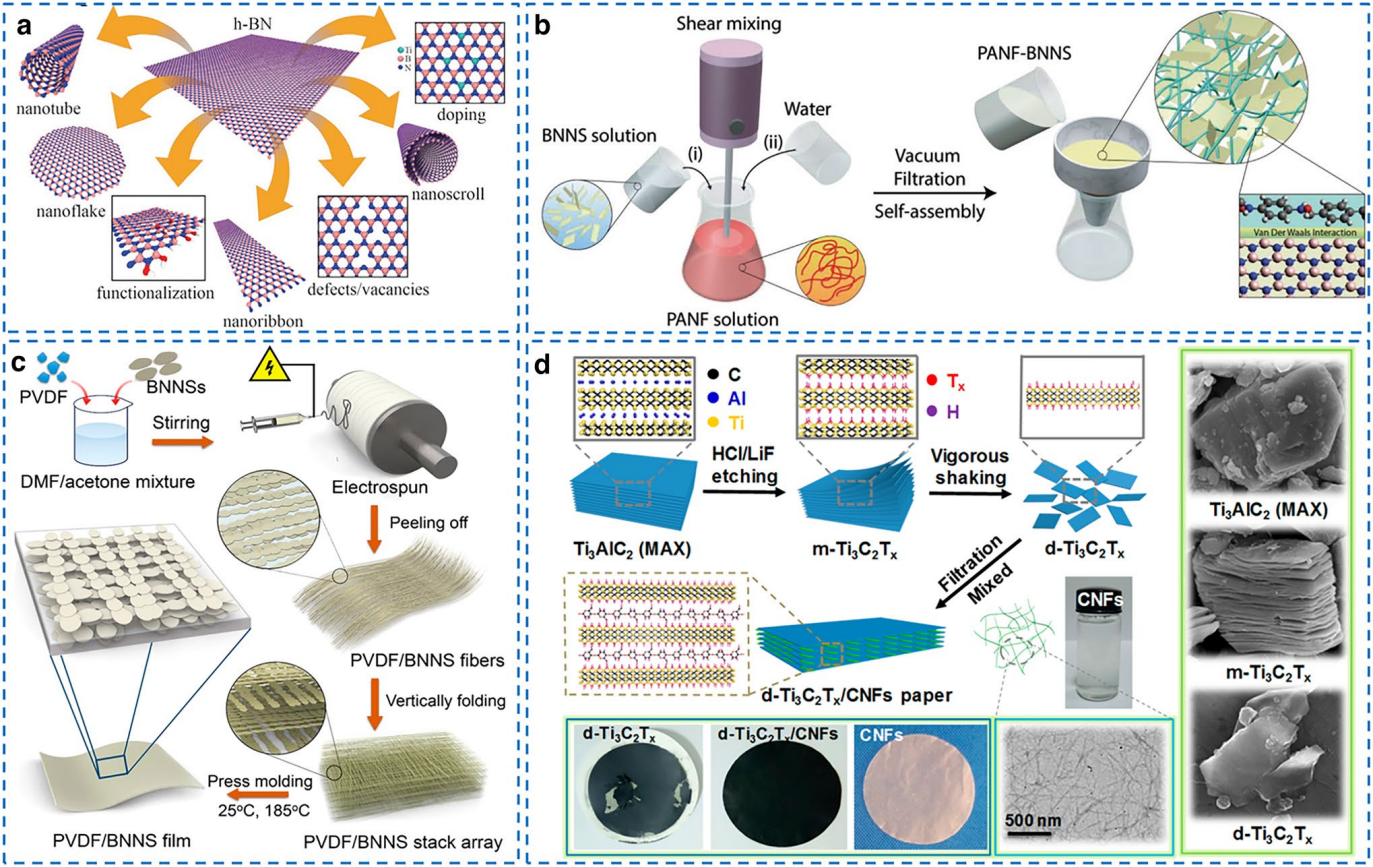
Fabrication of thermally conductive ceramic/polymer composite films with high in-plane *k*. **a** Possible configurations derived from BN. Reproduced with permission from Ref. [134]. Copyright 2021, Wiley–VCH. **b** Preparation process of the BNNS/PI nanofibers films using vacuum filtration method. Reproduced with permission from Ref. [138]. Copyright 2019, Wiley–VCH. **c** Preparation process of the BNNS/ PVDF films using electrospinning method. Reproduced with permission from Ref. [139]. Copyright 2019, American Chemical Society. **d** Preparation process of the MXene/NFC films using vacuum filtration method. Reproduced with permission from Ref. [140]. Copyright 2018, American Chemical Society

As shown in Fig. 9a, BN is very versatile and conformable in various geometric patterns, which can be exfoliated/transferred to BN nanosheets (BNNS), BN spherical nanoparticles, BN nanoribbons, BN nanoscroll, and BN nanotubes (BNNT) via ultrasonication, liquid exfoliation, plasma treatment and CVD method [134]. Using MD simulation, Zhu et al. [135] found that the order of thermal conductivity enhancing effectiveness of BN-based nanofillers is ranked as BNNS > BNNT > BN nanoparticle. Thus, BNNS fillers have been widely used to enhance the *k* of polymer. For example, Lu et al. [136] proposed a mechanochemical method to facilitate the exfoliation of bulk BN and covalent bond formation with PI chains, which strongly enhanced the interfacial affinity and in-plane orientation of BNNS filler in BN/PI films. The resultant films exhibited an in-plane *k* of 14.7 W (mK)^−1^ with 20 wt% BNNS filler. Similarly, Lin et al. [137] developed a microfluidization approach to obtain exfoliated BNNS with a high aspect ratio of 1500 and then utilized these BNNS to fabricate foldable BNNS/ PVA films with high in-plane *k* of 67.6 W (mK)^−1^.

Of note, owing to the stiffness and fragile property of ceramic fillers, it is troublesome to fabricate all-ceramic-based films. Thus, a polymer component is also needed to facilitate the uniform dispersion of ceramic fillers in solvents and to enhance the linking between adjacent ceramic fillers. Table 5 shows the summary of thermally conductive ceramic/polymer composite with high in-plane *k*. The ceramic/polymer composite films with high in-plane *k* and excellent insulating performance have been fabricated by many methods, including doctor blading [141], hot compression [142], solvent casting [143] and vacuum filtration [144]. Specifically, as shown in Fig. 9b, Ajayan et al. [138] fabricated a multifunctional film by integrating PI nanofibers with BNNS via vacuum-assisted LBL infiltration method, and the resulting films exhibited a high *k* of 2.4 W (mK)^−1^, good dielectric breakdown strength of 292 MV m^−1^ and high thermal stability (glass transition temperature of about 280 °C and degradation temperature of about 520 °C). As shown in Fig. 9c, Huang et al. [139] used electrospinning technique to prepare BNNS/polyvinylidene fluoride(PVDF)films with a high in-plane *k* of 16.3 W (mK)^−1^ and outstanding flexibility, in which BNNS fillers were aligned and interconnected along the in-plane direction of films. Similarly, Zhu et al. [145] reported a PVDF/BN films with intercalated sandwich structure fabricated by a LBL coating method, showing a high in-plane *k* of 18.4 W m^−1^K^−1^ and breakdown strength of 96.7 kV mm^−1^ at a filler content of 40 wt%. Moreover, Wang et al. [146] developed a sol–gel film conversion method to fabricate BN/poly(pophenylene benzobisoxazole) films with outstanding in-plane *k* of 214.2 W (mK)^−1^ with the help of poly(pophenylene benzobisoxazole) nanofiber and the resultant films showed plastic-like ductility of 80%, ultrahigh toughness of 100 MJ m^−3^ and high fatigue resistance.

**Table 5:** The summary of thermally conductive ceramic/polymer composite films

filler	Matrix	Fabrication method	Filler loading	In-plane *k* (W (mK)^−1^)	Measurement method	Flexibility and toughness	Refs.
BNNS	PI	Hot compression	12.4 vol%	4.25	LFT	–	2020 [142]
BN/GO	PI	Solvent casting	1 wt% GO and 20 wt% BN	11.20	LFT	–	2020 [143]
BN	PI	Mechanochemical method	20 wt%	14.7	LFT	Elongation at break of 25.9%	2021 [136]
Carbon nitride nanosheets	PI	Solvent casting	20 wt%	2.04	LFT	Withstanding folding deformations	2020 [147]
BNNS	SiO_2_@NFC	Vacuum filtration	7 wt%	10.88	LFT	Elongation at break of 10%	2021 [144]
BN	Poly(vinylidene fluoride)	Solvent casting	40 wt%	18.4	LFT	Breakdown strength of 96.7 kV/mm	2020 [145]
BN	Poly(p-phenylene benzobisoxazole) microfiber	Sol − gel film conversion	80 wt%	214.2	LFT	Elongation at break of 80%; toughness of 100 MJ/m^3^	2020 [146]
Nanodiamond@BNNS	PVA	Solvent casting	30 wt%	15.49	LFT	–	2020 [148]
BNNS	Ethylene–vinyl acetate copolymer (EVA)	Vacuum filtration	50 wt%	13.2	LFT	Withstanding folding different shapes; 5000 bending cycles	2020 [149]
BN	PVA	Vacuum filtration	90 wt%	120	LFT	–	2017 [150]
BN	NFC	Vacuum filtration	50 wt%	145.7	LFT	Withstanding folding deformations	2014 [18]
BNNS	PVA	Vacuum filtration	83 wt%	67.6	LFT	Withstanding folding different shapes; 2000 bending cycles	2021 [137]
BNNT	Aramid nanofiber + polyethyleneimine (PEI)	Vacuum filtration	15 wt%	9.91	LFT	Elongation at break of 12.8%;	2021 [151]
Carbon nitride nanosheets	PI	Vacuum filtration	20 wt%	2.04	LFT	Withstanding folding deformations	2020 [147]
MXene	PVA	Multilayered casting	19.5 wt%	4.57	LFT	Withstanding bending	2020 [152]
MXene	NFC	Vacuum filtration	30 wt%	22.43	LFT	Toughness of 1.0 MJ/m^3^; withstanding folding different shapes	2021 [153]
MXene/BNNS	NFC	Vacuum filtration	70 wt%	19.97	LFT	–	2021 [154]
BNNS@Al_2_O_3_	Silicone rubber	Shear coating	30 wt%	2.78	LFT	Elongation at break of 91%	2021 [114]

In comparison with BN fillers, other commonly used ceramic fillers, such as aluminum oxide (Al_2_O_3_) and silicon oxide (SiO_2_), show relatively lower intrinsic *k* [63], but have also been broadly adopted by industrial community because of their low cost [53, 155]. Recently, owing to the excellent conductivity, high charge storage capability and water dispersibility, MXene fillers have caught great enthusiasm in the fields of electromagnetic absorption, energy storage and electrocatalysis [85, 156–159]. Although MXene shows low intrinsic *k* compared with other two-dimensional fillers [152], MXene-based thermally conductive films have also been examined. The first-principle simulation demonstrated that the intrinsic *k* of MXene fillers is in the range of 54.3–104.7 W (mK)^−1^ [160]. Figure 9d shows the typical preparation process of MXene/NFC films via the vacuum filtration method. MXene/NFC films with an in-plane *k* of 22.43 W (mK)^−1^ [153], Mxene/BNNS/NFC films with an in-plane *k* of 19.91 W (mK)^−1^ [154], MXene/PVA films with an in-plane *k* of 4.57 W (mK)^−1^ [152] and GO/MXene films with an in-plane *k* of 26.49 W (mK)^−1^ [161] have been reported. However, the preparation of MXene involves harsh and toxic chemical etching routes and usually exhibits poor thermal stability and low intrinsic *k,* which greatly limited their industrial thermal management applications [162, 163].

### Comparison of Flexible Thermally Conductive Films

Table 6 gives the comparison of the characteristics, advantages and disadvantages of four kind of flexible thermally conductive films. One can see that all-carbon films show the highest *k*, while the disadvantages of all-carbon films is also noteworthy, including high cost, brittle, no electrical insulation and complicated fabrication process. The greatest advantage of intrinsically thermally conductive pure polymer films is no filler to need, while translating such superhigh *k* in nanofilms into bulk polymers presents a major challenge in practical fabrication, and the fabrication process of these films is too complicated. Thermally conductive carbon/polymer and ceramic/polymer composite films with advantages of low cost, high toughness and easy fabrication have present potential application in thermal management, while the low *k* greatly limited their thermal dissipation ability, especially for ceramic/polymer composite films.

**Table 6:** The comparison of characteristics, advantages and disadvantages of four kind of flexible thermally conductive films

Category	Characteristics	Advantages	Disadvantages	In-plane *k* (W (mK)^−1^)
Pure polymer films	The alignment of polymer chains through stretching or adjusting the molecules interactions of polymer	No fillers need; electrical insulation; excellent mechanical properties; low density	High cost; complicated fabrication process; low *k*	0.5–100
All-carbon films	Thermal annealing of GO films at high temperatures and hot compressing; PI graphitization at high temperatures	Promising *k*; high conductivity	High cost; brittle; no electrical insulation; complicated fabrication process	50–3000
Carbon/polymer composite films	Flexible polymer loaded with carbon-based fillers such as graphite and graphene	Low cost; easy fabrication; high toughness	No electrical insulation; high ITR at the filler/polymer interfaces	5–300
Ceramic/polymer composite films	Flexible polymer loaded with ceramic-based fillers such as BN and Al_2_O_3_	Low cost; easy fabrication; electrical insulation; high toughness; good antioxidant performance	Low *k*; high fillers loading need; high ITR at the filler/polymer interfaces	1–200

### Strategies to Reduce ITR

**Fig. 10:**
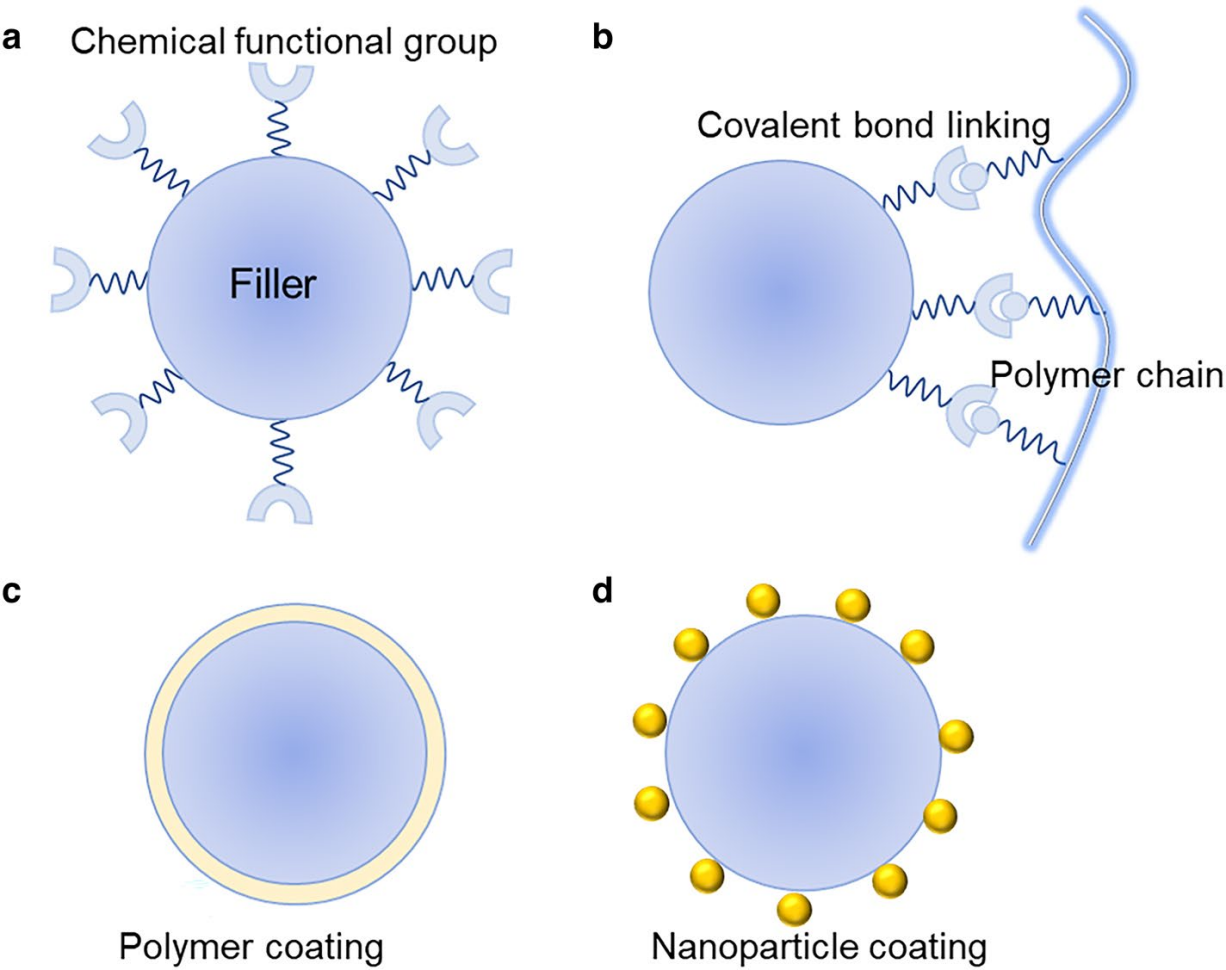
Effective methods to reduce the ITR. **a** chemistry modification of filler surface, **b** constructing covalent bonds between filler and polymer or filler and filler, **c** coating polymer layer on the surface of fillers, and **d** constructing “bridge” structure using nanoparticles

High ITR and strong phonon scattering at the interfaces (filler/filler, polymer/polymer and filler/polymer) are the main bottlenecks hindering the effective enhancement of *k* of thermally conductive materials [164, 165]. When the filler loading is low, the ITR between filler and polymer matrix is the dominating factor which influences the *k* of composites, while when the filler network structures are formed at high loadings, the primary influencing factor turns to the ITR between filler and filler [166]. Non-equilibrium Green’s function calculation results suggested that interfaces with strong interfacial bonding strength and smaller interfacial lattice mismatch give low ITR [167]. Table 7 shows the summary of previously reported works focusing on reducing ITR. Effective strategies to reduce ITR mainly include chemistry modification of filler surface [168], construction of covalent bond between filler/polymer and filler/filler [169, 170], coating polymer layer on the surface of fillers [171], and design of “bridge” structure between filler/polymer and filler/filler using nanoparticles [172]. Figure 10 presents the schematic presentation of these strategies.

**Table 7:** The summary of previously reported works to reduce ITR

Matrix	Filler	Strategy	Method	*k* enhancement (%)	Refs.
PVA	BN	Polymer coating	PDA coating	21	2015 [141]
PVA	BNNS	Polymer coating	PDA coating	23	2019 [171]
HDPE	BNNT	Polymer coating	PE coating	~ 30	2018 [173]
PS	BN	Polymer coating	PS coating	78	2017 [174]
NFC	BNNTS	Constructing “bridge” structure	AgNPs coating	62	2018 [175]
BC	GNPs	Constructing “bridge” structure	AgNPs coating	40	2020 [176]
Epoxy	GNPs	Constructing “bridge” structure	Al_2_O_3_ coating	~ 10	2016 [177]
NFC	BNNS	Chemistry modification of filler surface	Enhancing H-bonding interaction	98	2018 [168]
Epoxy	BNNS	Chemistry modification of filler surface	Enhancing interface compatibility	17	2014 [178]
Epoxy	GNPs	Chemistry modification of filler surface	Enhancing interface compatibility	30	2020 [179]
PI	RGO	Covalent bond linking	C-N–C bonds linking	30	2021 [169]
PI	GO	Covalent bond linking	C-N–C bonds linking	152	2013 [180]
NR	CNTs/BN	Covalent bond linking	C-N–C bonds linking	83	2020 [170]

Many studies have proved that rational chemistry modification of fillers including covalent and non-covalent functionalization can enhance the interface compatibility of filler/polymer and improve the dispersity of fillers, and the introduced chemical functions can effectively connect the vibrational phonon spectra of polymer to thermally conductive fillers (Fig. 10a) [179, 181]. For example, Ding et al. [182] studied the effect of functionalization degree of BNNS by isopropanol on the *k* of PI films and found that optimized interfacial interaction between filler/polymer is beneficial to the *k* improvement. Similarly, Bai et al. [183] used three divergent amines of ortho-phenylenediamine, meta-phenylenediamine and para-phenylenediamine to functionalize BN surface, signifying the significance of covalent interaction of -NH_2_ with BN as well as strong hydrogen bonding with thermoplastic polyurethane (TPU) matrix. The amine-functionalized BN filled TPU composites exhibited higher *k* than pristine BN filled counterparts.

However, MD simulation results showed that functionalized CNTs possessed lower *k* compared to pure CNTs, and the *k* decreased with the increase of functional group content [184]. Wong et al. [185] studied the *k* of BN monolayers covalently connected with -OH and -O(CH_2_)_4_CH_3_ groups and found that the intrinsic *k* of functional BN was significantly reduced with increasing loading of functional groups. Therefore, the key point for chemistry modification of fillers is how to simultaneously import desired functional groups but retain the intact crystal lattice of thermally conductive fillers [186]. More interestingly, Fu et al. [168] developed an edge-selective hydroxylation method to modify BNNS fillers, which simultaneously retained the perfect crystal lattice of fillers and enhanced the interface compatibility between NFC matrix and BNNS. The resultant BNNS-OH/NFC films showed a *k* enhancement of 98% compared to BNNS/NFC films.

Commonly used approaches to construct covalent bond connection mainly include a two-step method of filler activation with functional surface groups (e.g., -OH and -NH_2_), followed by the reaction with polymer chains [173] (Fig. 10b). For example, Gu et al. [169] used C-N–C bonds to connect PI matrix and RGO and improve the interface compatibility. When the filler loading was 15 wt%, the in-plane *k* of RGO- NH_2_/PI films reached up to 7.13 W (mK)^−1^, significantly higher than that of RGO/PI films at the same filler loading (5.5 W (mK)^−1^). Similarly, Lu et al. [170] reported a BN/CNTs/NR composite with the *k* of 1.34 W (mK)^−1^, and the BN and CNTs filler in the composites are connected by covalent bonds via the reaction of -COOH and -NH_2_ groups, which shows significant contribution to reducing filler/filler ITR and phonon scattering.

Coating nanoscale polymer thin film on the surface of fillers, including polydopamine (PDA) coating [141], PE coating [173], polystyrene (PS) coating [174], glycidyl methacrylate (GMA) coating [180] and polymethyl methacrylate (PMMA) coating [187], is another effective strategy to enhance the interfacial interaction between filler/polymer and enhance filler dispersion (Fig. 10c). For example, Tseng et al. [180] used glycidyl methacrylate (GMA) to graft-modify GO to fabricate g-RGO/PI films to enhance the compatibility between filler/polymer. When the amount of g-GO was 10 wt%, the in-plane *k* of g-RGO/PI films was improved to 0.81 W (mK)^−1^, much higher than 0.32 W (mK)^−1^ for GO/PI films with the same content of fillers. Owing to the strong π-π interaction between PDA and fillers, fillers such as BN, graphene and CNTs with conjugate structure, can be effectively modified via dopamine chemistry [141]. Thus, PDA coating on filler surface is quite effective to increase the interfacial compatibility between filler/polymer. For instance, Li et al. [171] fabricated a PDA@BNNS/PVA films with an in-plane *k* of 24.6 W (mK)^−1^, 23% higher than that of BNNS/PVA films, in which the PDA layer self-polymerized from dopamine effectively enhanced the interfacial interaction between filler/polymer.

Many studies have demonstrated that constructing “bridge” structure between fillers/matrix or filler/filler through nanoparticles was another effective strategy to reduce the thermal resistance and increase the contact area to transport heat. Thus, AgNPs [52, 141], copper nanowires [25], Cu microparticles [188], nanodiamond [148] and Al_2_O_3_ nanoparticles [177] have been used as the “bridge” units to enhance the *k* of films. MD simulation also demonstrated that intimate contact and high overlap area can cause significant reduction in ITR [189]. For example, Sun et al. [166] developed an effective method to reduce ITR in BNNS/NFC films through constructing “AgNPs-bridge” between BNNS fillers, and the ITR between BNNNs fillers was reduced to 4.6 × 10^–9^ m^2^K W^−1^ for AgNPs@BNNS/NFC films from 1.8 × 10^–8^ m^2^K W^−1^ for BNNS/NFC films. Besides, the “AgNPs-bridge” strategy has also been utilized in NFC/PVA [141], BNNTs/NFC [175] and BNNS/silicon carbide nanowires/PVA [190] composites to reduce ITR, and the *k* enhancement is in the range of 20–60% [141, 175, 190].

The effective medium theory [191], modified Hashin–Shtrikman model [192], Foygel model [193] and modified medium approximation [194] have been widely used to calculate the ITR by analyzing experimental data of composites. For example, Zeng et al. [135] used Foygel model to calculate the ITR in AgNPs@BNNS/AgNW/epoxy composites (18.4 × 10^–9^ m^2^K W^−1^) and BNNS/AgNW/epoxy composites (18.4 × 10^–9^ m^2^K W^−1^), and their studies demonstrated that the ITR was reduced by improving the contact area and interconnection between fillers. The used Foygel model is given by Eqs. (7) and (8) [27, 175, 195]:

7 $$k - k_{m} = k_{0} \left[ {\frac{{V_{f} - V_{c} }}{{1 - V_{c} }}} \right]^{\beta }$$

8 $${\text{ITR}} = \left( {k_{0} {\text{LV}}_{c}^{\beta } } \right)^{ - 1}$$

where *k*_*m*_*, k*_*0*_*, V*_*f*_*, V*_*c*_*, L* and *β* are the *k* of the polymer matrix, *k* of filler network, volume fraction of fillers, percolation threshold of composites, average length of filler and conductivity exponent dependent on the aspect ratio of fillers, respectively [196].

Wu et al. [191] used the effective medium theory to calculate the ITR in BP-CNTs/NFC composites (1.65 × 10^−9^ m^2^K W^−1^) and found that the ITR was decreased to 1/39 of that of pure CNTs owing to that an effective heat conduction pathway was constructed with the addition of amino-functionalized black phosphorene (BP-NH_2_) capable of bridging the CNTs nanosheets via covalent bonds. The model can be given by Eqs. (9) and (10) [191, 197]:

9 $$k_{{{\text{in}} - {\text{plane}}}} = k_{m} \frac{{2 + f\left[ {\frac{{k_{p} }}{{k_{m} }}\left( {1 + (\cos \theta )^{2} } \right)} \right]}}{{2 - f\left[ {\frac{{k_{p} h - k_{m} h - {\text{ITRk}}_{p} k_{{{\text{pm}}}} }}{{k_{p} h}}\left( {1 - (\cos \theta )^{2} } \right)} \right]}}$$

10 $$f = \frac{{w\rho_{m} }}{{w\rho_{m} + \left( {1 - w} \right)\rho_{p} }}$$

where *k*_*m*,_ *k*_in-plane_ and *k*_*p*_ are the *k* of the matrix, the composites in in-plane direction and the filler, respectively. *h* is the thickness of the composites, *f* is the volume fraction of the filler, *w* is the mass fraction of the filler, *ρ*_*m*_ is the density of the matrix, *ρ*_*p*_ is the density of the composites and $\theta$ is the orientation angle of filler in composites [191, 197].

## Applications

The fabrication of flexible thermally conductive films with high in-plane *k* has become the research frontier, while the commercial applications of these films are still in their early stages of development [198]. Flexible thermally conductive films are suitable for applications where in-plane conduction and flexibility are critical, such as flexible heat spreaders, personal thermal management, energy storage devices and electrocaloric cooling devices [29, 199].

### Flexible Heat Spreader

Flexible films with high in-plane *k* can be used as lateral heat spreaders which can cool local hot spots by transferring heat through the basal plane of the films. Films with higher in-plane *k* than through-plane *k* can be realized most of the heat preferentially dissipate from the hot resource along in-plane direction while not affecting the neighboring devices [112]. Copper and aluminum films have been used as conventional heater spreaders in many industrial fields [200]. However, high density and easy corrosion make them unfavorable for some special applications such as aerospace applications [127]. Thin graphite-based heat spreaders with low density are a promising passive thermal solution for hot spot elimination on enclosure surfaces of electronics owing to their high in-plane *k* and low cost [200]. Flexible NG films and graphitized PI film are typical industrial heat spreaders which have been used in various electronics including touch panels, iPad, smartphones and LED modules [103].

**Fig. 11:**
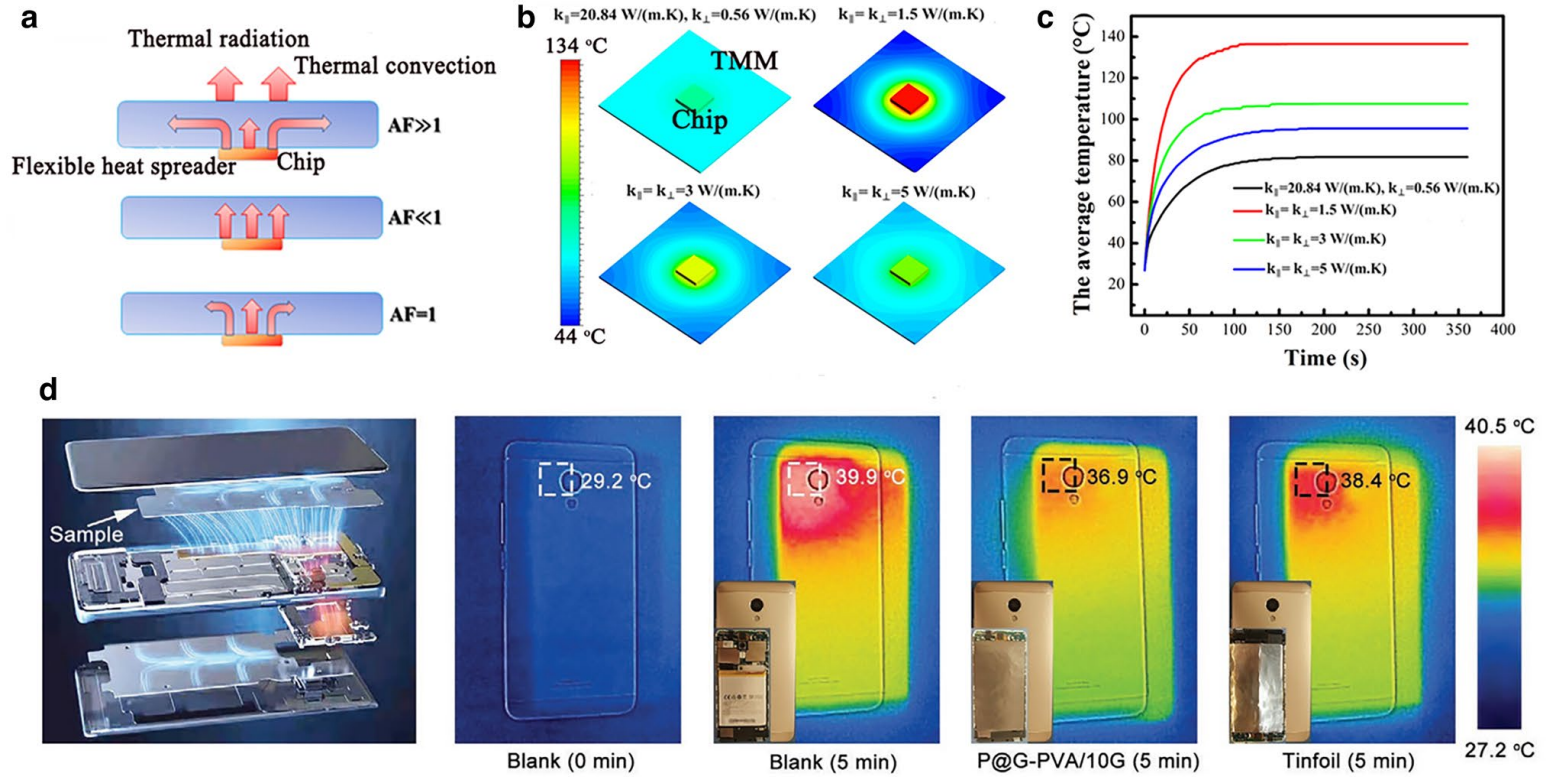
**a** Schematic illustration of the thermal conduction of thermally conductive films with different anisotropic factors (AF, a ratio of in-plane *k* to through-plane *k*), **b** the modeling of the temperature at *t* = 360 s when using flexible heat spreader with different *k*, **c** the average temperature change of the chip with working time when using flexible heat spreader with different *k*. Reproduced with permission from Ref. [128]. Copyright 2019, American Chemical Society. **d** The thermal infrared images of smartphones under different working conditions when using phenylphosphonic acid@GNPs/PVA film and tinfoil as flexible heater spreader to cool smartphone. Reproduced with permission from Ref. [115]. Copyright 2021, Wiley–VCH

In our previous work, the application of the thermally conductive films as flexible heat spreaders and electric substrates has been clarified by combining experimental data and finite element analysis simulation, confirming the significance of the in-plane *k* in thermal management of electronics [128]. Figure 11a shows that when the size of chip is smaller than that of flexible heat spreaders, the films with strong *k* anisotropy can spread the heat from a small hot spot of the chips to a larger area to make more efficient use of the thermal convection and radiation from the surface of heat spreader. As shown in Fig. 11b–c, the simulation results demonstrated that the equilibrium temperature of the chip using the anisotropic RGO/NR films with in-plane *k* of 20.84 W (mK)^−1^ and through-plane *k* of 0.56 W (mK)^−1^ is about 80 °C, which has dropped by about 15, 27 and 56 °C in comparison with the cases using isotropic films with *k* of 5, 3 and 1.5 W (mK)^−1^, respectively [128].

Owing to the higher *k* of RGO films than that of graphite films, RGO heat spreaders show higher heat dissipation ability, and it has been reported that commercial RGO films have been used as flexible heat spreaders in the smartphone of “Mate 20” [87]. Recently, Song et al. [115] used phenylphosphonic acid@GNPs/PVA film as flexible heater spreader to cool smartphone. As shown in Fig. 11d, without using the heater spreader, the backside temperature of the smartphone quickly rose from 29.2 to 39.9 °C after running large game program for 5 min. After integrating thermally conductive as-fabricated films and commercial tinfoil, the backside temperature of the smartphone was reduced to 36.9 and 38.4 °C, respectively. Similarly, Kim et al. [105] found that the temperature of high-power LED was 58 °C when using exfoliated graphene fluoride film as the heat spreader, much lower than that of commercial PI film of 118 °C. Lin et al. [137] used BNNS/PVA films as the heat spreader of high-power LED modules, and the results showed that BNNS/PVA films exhibited superior cooling efficiency compared to commercial flexible copper films. Moreover, Reis et al. [201] studied the heat dissipation ability of graphite films as heat spreader by attaching to acrylonitrile–butadiene–styrene copolymer (ABS) substate and found that both graphite films with in-plane *k* of 425 and 1000 W (mK)^−1^ offered promising heat spreading and hot spot reduction abilities. Their results showed that the heat dissipation ability of graphite films was significantly better than that of copper and aluminum spreaders. In addition, RGO films and PI films can be hot-pressed together to fabricate flexible print circuit board with sandwich structure, which can reduce the chip temperature to be 10 ℃ lower than traditional print circuit board [95].

### Personal Thermal Management

Human beings are sensitive to humidity and temperature of outside environment, and human body feels comfortable when the temperature is in the range of 23 to 26 °C and the relatively humidity is in the range of 30 to 55% [202]. Flexible thermally conductive films with excellent radiative heat dissipation can also be used in personal thermal management to adjust the temperature and humidity of body [203]. Wearable textiles with high *k* and excellent cooling effect can provide the building occupants with thermal comfort in hot weather or working surroundings via heat exchange between human body and environment [204, 205].

**Fig. 12:**
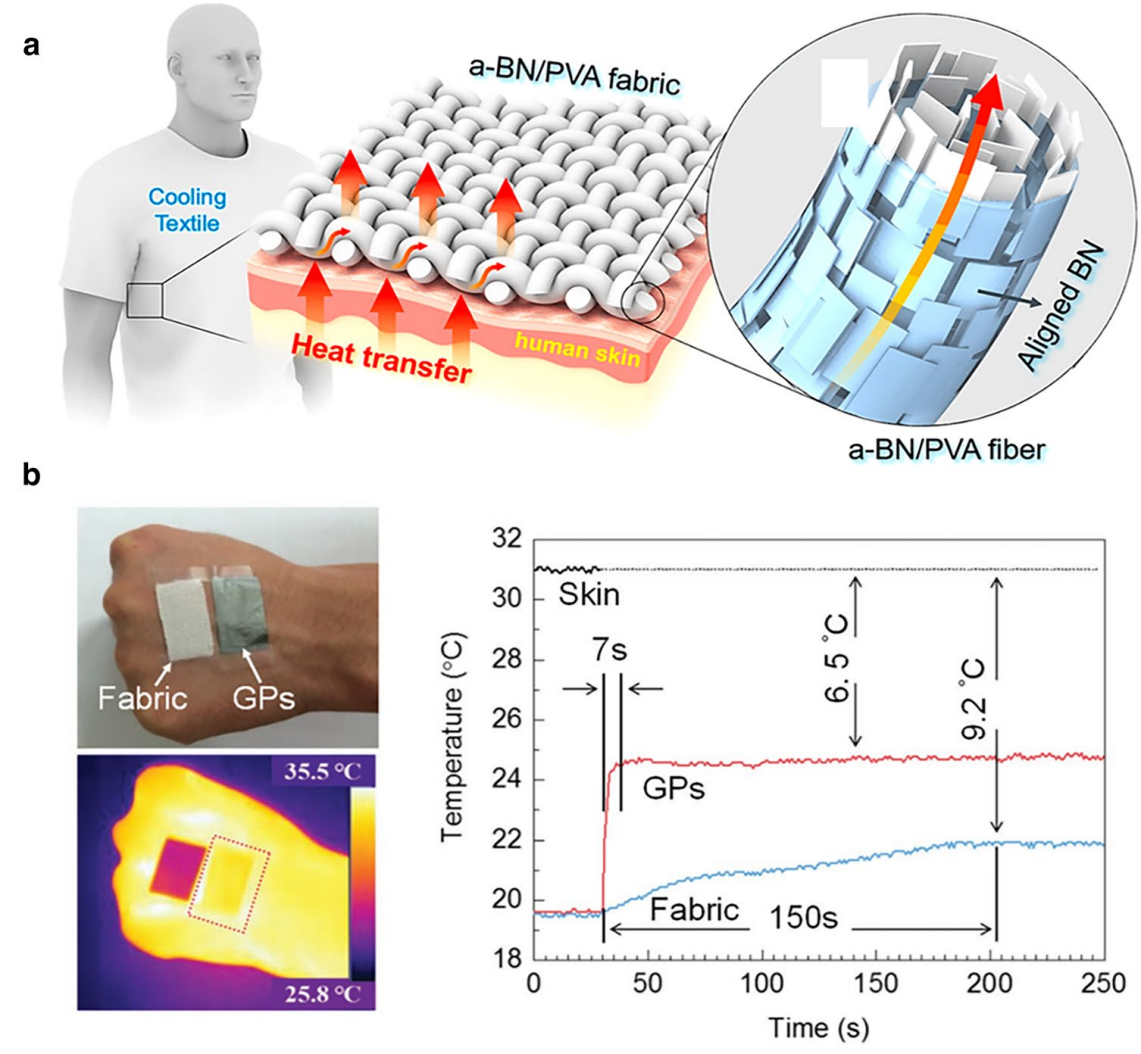
**a** Schematic illustration of BNNS/PVA textile with high in-plane *k* used in personal thermal management Reproduced with permission from Ref. [204]. Copyright 2017, American Chemical Society. **b** Photograph, infrared image, and the corresponding response time and heating transfer of normal cotton fabric and graphene film placed on skin. Reproduced with permission from Ref. [88]. Copyright 2017, Wiley–VCH

Considerable efforts of developing thermal regulation textiles with high in-plane *k*, such as graphene-coated polyester textiles [206], CNT-coated cotton textiles [207], AgNW-coated cotton textiles [208], BN/PI textile [209] and AgNW nonwoven/PI [210], have been made recently. For example, as presented in Fig. 12a, Hu et al. [204] reported a BNNS/PVA textile with a high in-plane *k* for efficient personal thermal cooling, and the textile prepared by 3D printing technology can effectively remove the body-generated heat from the skin to the outside, showing a 55% enhancement in personal cooling efficiency compared to commercial cotton textiles. Similarly, as presented in Fig. 12b, Wang et al. [88] found that pure RGO films can also be used as high-efficiency personal thermal management materials to ensure safety and comfort of human body in complex environments without compromising breathability. The temperature gap between skin and RGO films showed a 2.7 °C reduction compared to normal fabrics.

### Energy Storage Devices

A large amount of heat is generated during the charging/discharging process of electrochemical energy storage devices, which causes a rapid degradation of capacity and thermal runaway [13, 211, 212]. When Li-ion battery and supercapacitor are operated at high temperature, the softening of polymer separator is possible to cause electrical short circuit [213]. Thus, thermally conductive films can also be used as the thermal management materials in energy storage devices to dissipate excess heat [13, 214]. The research results demonstrated that BNNS composite membrane separator for Li-Ion battery can withstand an operating temperature up to 150 °C [213, 214]. For example, Hu et al. [215] developed a thermally conductive separator coated by BNNS to enhance the thermal stability of Li-Ion battery. A more homogeneous thermal distribution and uniform deposition/striping of Li was achieved by incorporating BNNS, reducing the risk of dendritic growth of Li and enhancing cycling performance. Similarly, Ajayan et al. [138] utilized BNNS/polyaramid nanofibers films with high *k*, high microporosity and good wettability with organic solvents, as high-temperature Li-ion battery separator, and the results showed that cells presented no substantial loss in areal capacity after working at 120 °C for 5 cycles.

### Electrocaloric Cooling Devices

Electrocaloric cooling devices, enabled by the discovery of the giant electrocaloric effect in dielectrics, represents a zero-global-warming-potential, environment-benign cooling alternative [216]. The low *k* of ferroelectric polymer such as poly(vinylidenefluoride-co-trifluoroethylene) (P(VDF-TrFE)) hinders their electrocaloric cooling property. Thus, Wang et al. [217] used BNNS to improve the dielectric breakdown strength and *k* of ferroelectric polymer-based pyroelectric films for electrocaloric cooling refrigerators, and found that higher in-plane *k* was helpful to achieve high cooling efficiency and colling power density of electrocaloric cooling devices. The introduction of thermally conductive nanofillers into ferroelectric polymer endowed the films with anisotropic *k*, which could facilitate the thermal transfer between electrocaloric cooling materials in one direction and suppress the conduction loss in other directions [216].

## Conclusions and Outlooks

In this review, we survey the emerging research activities concerning flexible thermally conductive films, including intrinsic polymer films and polymer composite films with ultrahigh in-plane *k*, and their heat transfer mechanism and potential applications. Impressively, pure UHMWPE films with metal-like *k* of 63 W (mK)^−1^, and RGO films with ultrahigh *k* of 1940 W (mK)^−1^ have been fabricated. Compared with common thermally conductive materials, these thermally conductive films exhibited promising in-plane *k*, excellent flexibility, low thickness and outstanding mechanical strength, presenting great application potential in next-generation devices, such as on-skin electronics, personal thermal management and energy storage devices.

Despite these great endeavors, several issues need to be considered for further development of the state-or-the-art thermally conductive films. (1) UHMWPE nanofiber/nanofilms with superhigh *k* have been achieved. However, translating such superhigh *k* in nanofiber/nanofilms into bulk polymers presents a major challenge in practical fabrication. Thus, further optimization of the synthesis and processing of intrinsically thermally conductive materials are required to fabricate bulk polymer films with large thickness. (2) The low *k* of thermally conductive polymer composite films still limited their practical application. In addition to reducing ITR and constructing effective thermal transport pathways, improving the quality of fillers (crystal structure, crystallization degree, defects) is another strategy to enhance the *k* of composite, which has been ignored. Therefore, more attention should be paid to the synthesis of thermally conductive fillers with high quality to reduce the defects and impurities which greatly degrade the intrinsic *k* of fillers. (3) Although various thermal technologies have been developed, a standardization agreement among the international scientific community and industrial community on how to measure in-plane *k* of films is also needed. (4) Considering practical application requirement of thermally conductive materials, in addition to *k* value, the electrical insulation, coefficient of thermal expansion, thermal stability, flame resistance and fatigue resistance are well-advised to be considered. (5) Artificial intelligence technologies such as the machine learning method can be conducted to study the design rules of high-performance thermally conductivity films, which can significantly reduce labor costs and material costs and shorten the development cycle. (6) The gap between laboratory-scale proof-of-concept demonstration and industrial application needs to be filled. The vacuum-assisted filtration is the most widely used method to fabricate flexible thermally conductivity films, and the promising results have been achieved in the enhancement of *k*, but the size limitation from this method still overshadows their further industrial-scale applications.
